# 5-Substituted-furan-2(3*H*)-ones in [8 + 2]-Cycloaddition with 8,8-Dicyanoheptafulvene

**DOI:** 10.1021/acs.joc.2c00101

**Published:** 2022-03-29

**Authors:** Marta Romaniszyn, Lesław Sieroń, Łukasz Albrecht

**Affiliations:** † Institute of Organic Chemistry, Department of Chemistry, Faculty of Chemistry, Lodz University of Technology, Zeromskiego 116, 90-924 Łódź, Poland; ‡ Institute of General and Ecological Chemistry, Faculty of Chemistry, Lodz University of Technology, Żeromskiego 116, 90-924 Łódź, Poland

## Abstract

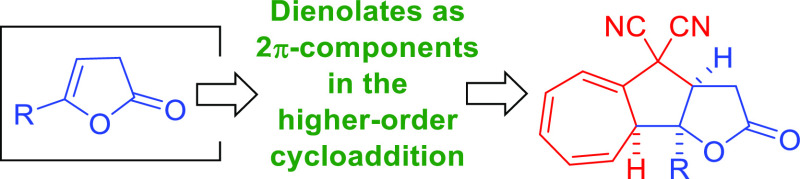

This study demonstrates the use of organocatalytic Brønsted base activation of 5-substituted-furan-2(3*H*)-ones to generate 2π-components for the diastereoselective [8 + 2]-cycloaddition involving 8,8-dicyanoheptafulvene as an 8π-component. The use of dienolates in a higher-order cycloaddition reaction leads to the formation of biologically relevant polycyclic products bearing a γ-butyrolactone structural motif, thus broadening the synthetic potential of Brønsted base activated higher-order cycloadditions.

## Introduction

**Scheme 1:**
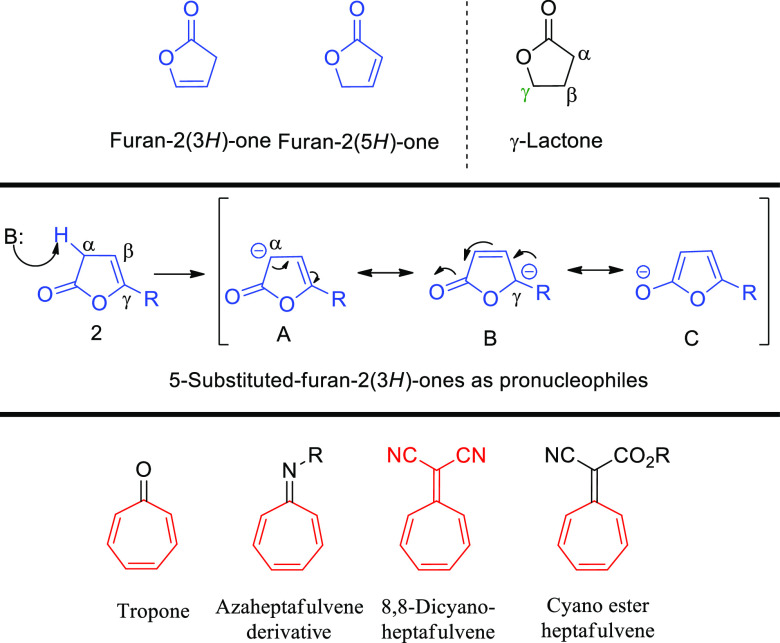
5-Substituted-furan-2(3*H*)-ones and Tropone Derivatives in Organic Synthesis

Lactones and their unsaturated derivatives—butenolides, are very interesting groups of chemical compounds. Naturally occurring lactones and their derivatives are present in over 13,000 natural products and have a broad spectrum of biological properties, making them a valuable synthetic target in the modern organic chemistry.^1^ Due to the great importance of lactones and their derivatives in the chemical and pharmaceutical environments, various methods of their synthesis are widely explored.^2^ Unsaturated β,γ- or α,β-butenolides [furan-2(3*H*)-ones or furan-2(5*H*)-ones] due to their high availability and reactivity constitute attractive synthons for the enantio- and diastereoselective synthesis of many biologically relevant structures containing γ-lactone rings (Scheme 1, top).^3^ In particular, 5-substituted-furan-2(3*H*)-ones deserve special attention because they are a useful group of pronucleophiles which undergo deprotonation from the α-position under organocatalytic conditions, thus transforming them into synthetically relevant dienolates with a vinylogous reactivity. This feature was successfully employed in various types of organic transformations with nucleophilic properties manifested from either α-^4^ or γ-position (Scheme 1, middle).^3,5^

Heptafulvenes and their hetero-analogues are another group of compounds with interesting properties and applications (Scheme 1, bottom). The presence of conjugated double bonds in their structure allows for synthetic applications in various types of cycloaddition reactions, leading to complex molecules that often have interesting biological activities. The cycloheptatriene ring can be found in many natural products and compounds relevant for life-science industry.^6^ Recently, their usage in organocatalytic higher-order cycloadditions has emerged providing access to complex polycyclic structures often difficult to prepare via classical synthetic approaches.^7,8^

**Scheme 2:**
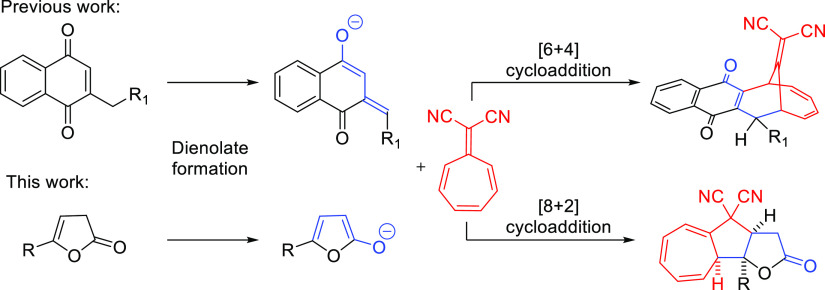
Dienolates as Higherenophiles in Higher-Order Cycloadditions—Previous Results and the Aim of the Present Studies

In 2019, our research group demonstrated the application of 2-benzyl-1,4-naphthoquinones as a novel group of 4π-components in the organocatalytic [6 + 4]-cycloaddition with 8,8-dicyanoheptafulvene (Scheme 2).^9^ A distinctive feature of the strategy was the transformation of these substrates into the corresponding dienolates under mild, organic Brønsted base catalytic conditions and their application as higherenophiles in the higher-order cycloaddition. Given the promising synthetic potential of such an approach, studies on the development of a new higher-order cycloaddition proceeding under Brønsted base catalysis were undertaken (Scheme 2). As the ability of 5-substituted-furan-2-(3*H*)-ones to provide the corresponding dienolates under basic conditions is well recognized, this group of compounds was selected as potential 2π-component precursors for the studies. It was anticipated that they should undergo higher-order cycloaddition with 8,8-dicyanoheptafulvene leading to the formation of polycyclic [8 + 2] cycloadducts as possible products. However, at the outset of our studies, the control of periselectivity as well as stereoselectivity of the process was of major concern.

Herein, we present our studies on the development of highly peri- and diastereoselective higher-order cycloaddition between 5-substituted-furan-2(3*H*)-ones and 8,8-dicyanoheptafulvene. The reaction was realized under Brønsted base catalysis providing polycyclic γ-lactone derivatives bearing cycloheptatriene structural motifs. Our approach benefits from operational simplicity using readily available substrates and a simple organocatalyst, providing structurally interesting products containing valuable functional groups with the potential biological activity in a diastereoselective manner.

## Results and Discussion

**Scheme 3:**
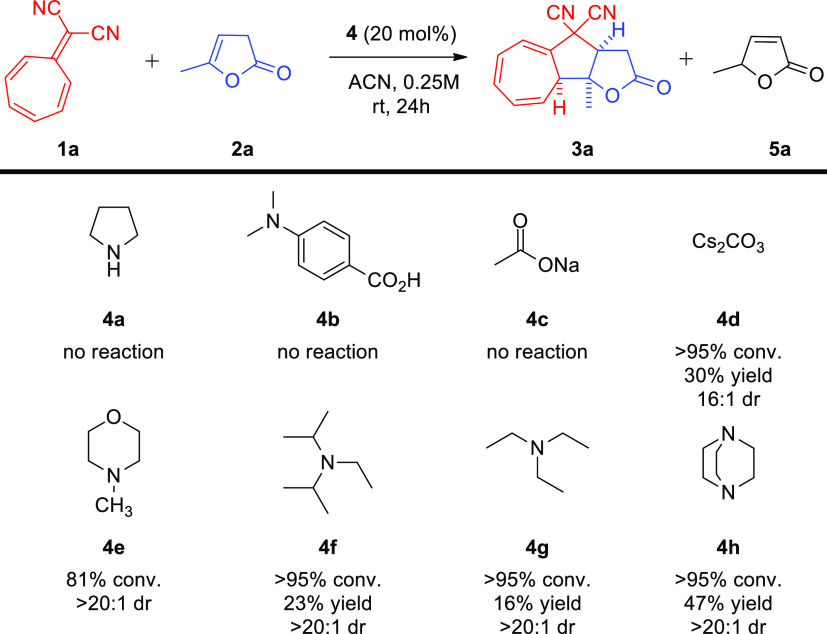
5-Substituted-furan-2-(3*H*)-ones **2** in the [8 + 2]-Cycloaddition with 8,8-Dicyanoheptafulvene **1a**—the Selection of Brønsted Base Catalyst

Optimization studies were carried out using commercially available 5-methylfuran-2-(3*H*)-one **2a** and 8,8-dicyanoheptafulvene **1a** as model reactants. Initially, studies were focused on finding an appropriate base to promote the designed reaction (Scheme 3). Due to the fact that lactone **2a** isomerized under the basic reaction conditions, experiments were performed utilizing 2-fold excess of **2a** in order to improve the yield of the cycloaddition. A number of organic and inorganic bases were tested for their ability to promote the reaction. In the presence of either pyrrolidine **4a**, 4-(dimethylamino)benzoic acid **4b**, or sodium acetate **4c** no formation of product **3a** was observed. Utilization of cesium carbonate **4d** induced the desired reactivity, but the cycloaddition proceeded in low yield and with moderate diastereoselectivity. Importantly, the use of tertiary amines **4e–h** improved both the conversion and diastereoselectivity of the process with DABCO **4h** providing the best results.

Subsequently, the solvent screening was performed (Table 1, entries 1–7). Unfortunately, none of the solvents tested improved the results despite prolonged reaction times. Therefore, the influence of temperature was investigated. Neither decrease (Table 1, entry 8) nor increase (Table 1, entry 9) of the temperature was beneficial for the developed higher-order cycloaddition and the results remained unsatisfactory. Finally, the change of the reaction concentration provided a positive effect on the efficiency of the process maintaining a high diastereomeric ratio (Table 1, entries 10–11). The highest yield was observed using a concentration of 0.125 M, thus indicating final reaction parameters (Table 1, entry 11). Importantly, the possibility to perform the reaction on a 1 mmol scale was also investigated with comparable results obtained (Table 1, entry 12).

**Table 1:**
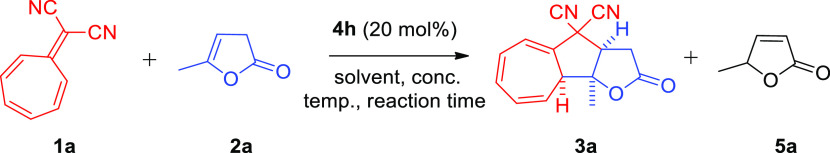
5-Substituted-furan-2(3*H*)-ones **2** in the [8 + 2]-Cycloaddition with 8,8-Dicyanoheptafulvene **1a**—Optimization Studies^a^

entry	solvent	reaction time [days]	conv. [%]^b^	dr^c^
1	CHCl_3_	3	14	7:1
2	CH_2_Cl_2_	3	53	13:1
3	toluene	3	19	
4	trifluorotoluene	3	17	9:1
5	2-methyltoluene	3	51	14:1
6	1,4-dioxane	3	60	6:1
7	THF	3	>95 (36)	12:1
8^d^	CH_3_CN	4	>95 (44)	>20:1
9^e^	CH_3_CN	1	>95 (48)	15:1
10^f^	CH_3_CN	1	>95 (62)	>20:1
11^g^	CH_3_CN	1	>95 (67)	>20:1
12^h^	CH_3_CN	1	>95 (68)	>20:1

a Unless otherwise stated, reactions performed on a 0.05 mmol scale using **1a** (1.0 equiv), **2a** (2 equiv), and catalyst **4h** (20 mol %) for 1–4 days at room temperature and 0.2 mL of the corresponding solvent.

b Conversion was determined by ^1^H NMR of a crude reaction mixture. In parentheses the isolated yield is given.

c Determined by ^1^H NMR of a crude reaction mixture.

d Reaction performed at 5 °C.

e Reaction performed at 40 °C.

f Reaction performed using CH_3_CN (0.1 mL).

g Reaction performed using CH_3_CN (0.4 mL).

h Reaction performed on a 1 mmol scale.

**Scheme 4:**
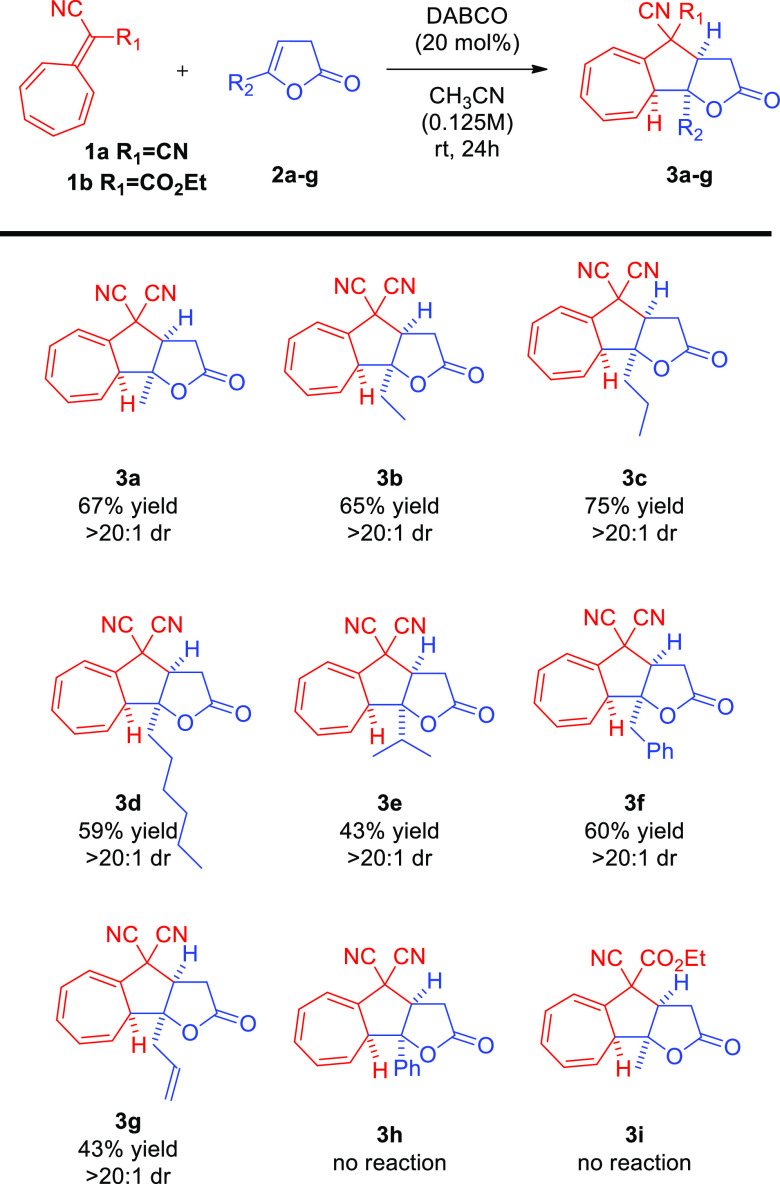
5-Substituted-furan-2(3*H*)-ones in the [8 + 2]-Cycloaddition with 8,8-Dicyanoheptafulvene—Scope Studies

Having optimized the conditions for the developed reaction, its scope and limitations were investigated (Scheme 4). Furan-2(3*H*)-ones **2a–g** with various substituents in the γ-position were reacted with 8,8-dicyanoheptafulvene **1a**. All higher-order cycloadditions proceeded in a highly diastereoselective manner. Furthermore, the change of length of the carbon chain in the γ-position of substrates **2a–c** had a positive effect on the efficiency of the reaction (Scheme 4, products **3a–c**). However, this trend was not continued with significant lengthening of the alkyl chain (Scheme 4, product **3d**). In the case of furan-2(3*H*)-one **2e** bearing more sterically demanding *i*-propyl group, a lower yield was observed (Scheme 4, product **3e**). The reaction proved unbiased toward the introduction of functional groups (double bond or phenyl ring) in the carbon chain in the γ-position of **2f–g** as demonstrated in the synthesis of **3f–g** (Scheme 4, products **3f–g**). To further broaden the scope of the developed method, 5-phenylfuran-2(3*H*)-one **2h** was employed. Unfortunately, no product formation was observed under established conditions. Attempts to re-optimize reaction conditions with **2h** were unsuccessful. Endeavors to expand the scope of the method by the use of ethyl 2-cyano-2-(cyclohepta-2,4,6-trien-1-ylidene)acetate **1b** were also unsuccessful as the formation of the product **3i** was not observed.

**Scheme 5:**
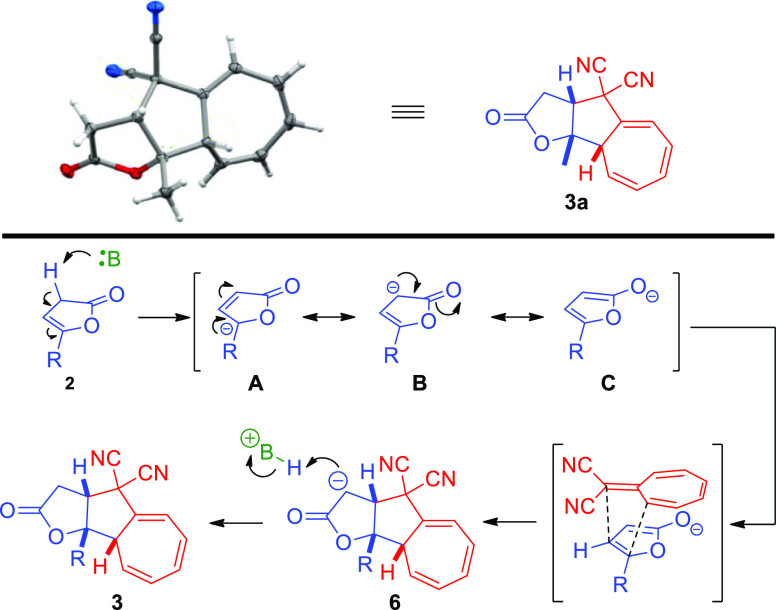
5-Substituted-furan-2(3*H*)-ones **2** in the [8 + 2]-Cycloaddition with 8,8-Dicyanoheptafulvene **1a**—Mechanistic Considerations

The relative configuration of **3a** was unambiguously confirmed by the single-crystal X-ray analysis (see the Supporting Information for further information). Relative configurations of the remaining cycloadducts **3b–g** were assigned by analogy (Scheme 5, top). Given the configurational assignments, a plausible mechanism and stereochemical model of the higher-order cycloaddition was proposed (Scheme 5, bottom). It is postulated that in the first step the organic base deprotonates 5-substituted-furan-2(3*H*)-one **2** from its α-position, therefore generating a dienolate represented by three resonance structures **A–C**. The dienolate that acts as a higherenophile in the developed cycloaddition undergoes cycloaddition with higherene **1a** to give **6**. Protonation of the α-position in the lactone ring leads to the formation of a final product **3**.

**Scheme 6:**
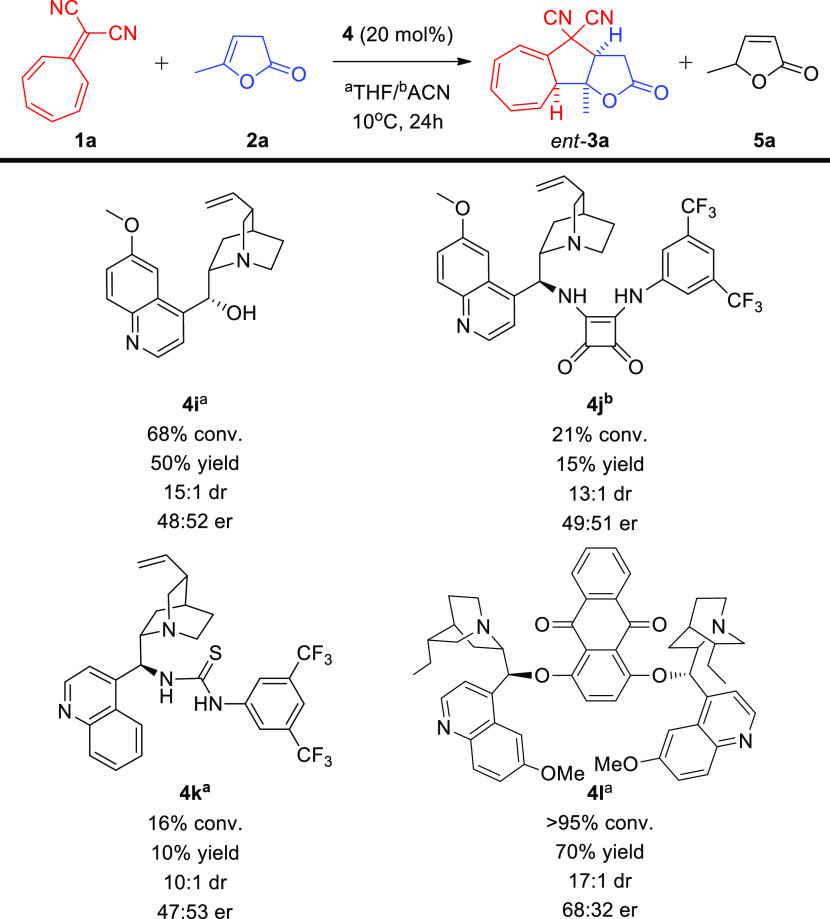
5-Substituted-furan-2(3*H*)-ones **2** in the [8 + 2]-Cycloaddition with 8,8-Dicyanoheptafulvene **1a**—Enantioselective Approach

Attempts to perform the developed higher-order cycloaddition in an enantioselective manner were also undertaken (Scheme 6). Therefore, a number of chiral organic bases were tested in order to evaluate their ability to induce asymmetry in the studied transformation. The use of the simplest quinine **4i** as a catalyst gave unsatisfactory results, both in terms of yield as well as in the diastereoselectivity and most importantly enantioselectivity of the process. Similar results concerning selectivity of the process, but even worse efficiency of the cycloaddition were obtained, when quinine squaramide derivative **4j** was used. Thiourea **4k** derived from cinchonidine also did not promote the reaction in an enantioselective manner. The best result was obtained when a dimeric catalyst **4l** was applied—conversion and yield of the cycloaddition were at satisfactory level, but diastereoselectivity as well as enantioselectivity still remained low.

## Conclusions

In conclusion, the application of 5-substituted-furan-2(3*H*)-ones **2** under organocatalytic conditions led to the formation of the active dienolate which acted as a 2π-component in the [8 + 2]-cycloaddition with 8,8-dicyanoheptafulvene **1a** as an 8π-component. It was possible to obtain cycloadducts **3a–g** efficiently and in highly diastereoselective manner. As a continuation of our previous work, the above research managed to expand the application of organocatalytically activated dienolates in the higher-order cycloadditions.

## Experimental Section

### General Information

Unless otherwise noted, analytical grade solvents and commercially available reagents were used without further purification. NMR spectra were acquired on a Bruker Ultra Shield 700 instrument, running at 700 MHz for ^1^H and 176 MHz for ^13^C, respectively. Chemical shifts (δ) are reported in ppm relative to residual solvent signals (CDCl_3_: 7.26 ppm for ^1^H NMR, 77.16 ppm for ^13^C NMR). Mass spectra were recorded on a Bruker Maxis Impact quadrupole time-of-flight spectrometer using electrospray (ES+) ionization (referenced to the mass of the charged species). Analytical thin layer chromatography was performed using pre-coated aluminum-backed plates (Merck Kieselgel 60 F254) and visualized by ultraviolet irradiation. Silica gel (Silica gel 60, 230–400 mesh, Fluka) was used for flash chromatography. 5-Methyl-furane-2(3*H*)-one **2a** is a commercially available compound.

#### 2-(Cyclohepta-2,4,6-trien-1-ylidene)malononitrile (**1a**)

Compound **1a** was synthesized according to the literature procedure^11^ as red needles in 75% yield (115.6 mg). Analytical data were in accordance with the literature.

#### Ethyl 2-Cyano-2-(cyclohepta-2,4,6-trien-1-ylidene)acetate (**1b**)

Compound **1b** was synthesized according to the literature procedure^11^ as a red solid in 70% yield (253.54 mg). Analytical data were in accordance with the literature.

#### 5-Ethylfuran-2(3*H*)-one (**2b**)

Compound **2b** was synthesized according to the literature procedure^12^ as a colorless oily liquid in 20% yield (23.8 mg). Analytical data were in accordance with the literature.

#### 5-Propylfuran-2(3*H*)-one (**2c**)

Compound **2c** was synthesized according to the literature procedure^13^ as a colorless oily liquid in 45% yield (20.1 mg). Analytical data were in accordance with the literature.

#### 5-Hexylfuran-2(3*H*)-one (**2d**)

Compound **2d** was synthesized according to the literature procedure^13^ as a yellow oily liquid in 40% yield (22.9 mg). Analytical data were in accordance with the literature.

#### 5-Isopropylfuran-2(3*H*)-one (**2e**)

Compound **2e** was synthesized according to the literature procedure^14^ as a light-yellow oily liquid in 41% yield (20.5 mg). Analytical data were in accordance with the literature.

#### 5-Benzylfuran-2(3*H*)-one (**2f**)

Compound **2f** was synthesized according to the literature procedure^13^ as a light-yellow oily liquid in 7% yield (18.1 mg). ^1^H NMR (700 MHz, CDCl_3_): δ 7.48–7.10 (m, 5H), 5.07–5.04 (m, 1H), 3.61 (q, *J* = 2.2 Hz, 2H), 3.17 (q, *J* = 2.4 Hz, 2H). ^13^C{^1^H} NMR (176 MHz, CDCl_3_): δ 176.6, 156.1, 135.3, 129.2 (2C), 128.8 (2C), 127.2, 99.8, 35.0, 34.1. HRMS (ESI-TOF) *m*/*z*: [M + H]^+^ calcd for C_11_H_10_O_2_H, 175,0759; found, 175.0758.

#### 5-Allylfuran-2(3*H*)-one (**2g**)

Compound **2g** was synthesized according to the literature procedure^13^ as a light-yellow oily liquid in 30% yield (19.1 mg). ^1^H NMR (700 MHz, CDCl_3_): δ 5.83 (ddt, *J* = 16.9, 10.1, 6.7 Hz, 1H), 5.23–5.19 (m, 1H), 5.19–5.17 (m, 1H), 5.15 (tt, *J* = 2.3, 1.5 Hz, 1H), 3.18–3.17 (m, 2H), 3.07–3.04 (m, 2H). ^13^C{^1^H} NMR (176 MHz, CDCl_3_): δ 176.7, 155.4, 131.2, 118.9, 99.1, 34.1, 32.8. HRMS (ESI-TOF) *m*/*z*: [M + H]^+^ calcd for C_7_H_8_O_2_H 125, 0603; found, 125.0603.

#### 5-Phenylfuran-2(3*H*)-one (**3h**)

Compound was synthesized according to the literature procedure^13^ as an orange solid in 65% yield (104,0 mg). Analytical data were in accordance with the literature.

#### General Procedure for the Synthesis of Compounds **3a–g**

In an ordinary 4 mL glass vial, equipped with a magnetic stirring bar and a screw cap, 5-substituted-furane-2(3*H*)-one **2** (2.0 equiv, 0.2 mmol), 8,8-dicyanoheptafulvene **1a** (1.0 equiv, 0.1 mmol, 15.42 mg), and catalyst **4h** (0.02 equiv, 0.02 mmol, 2,24 mg) were dissolved in acetonitrile (0.8 mL) and stirred for 24 h at room temperature. The reaction mixture was directly subjected to flash chromatography on silica gel (eluent = hexanes/ethyl acetate, 8:1) to obtain pure product **3**.

#### (3aR*,9aS*,9bR*)-9*b*-Methyl-2-oxo-3,3*a*,9*a*,9*b*-tetrahydroazuleno[1,2-*b*]furan-4,4(2*H*)-dicarbonitrile (**3a**)

Following the general procedure, pure product **3a** was isolated after flash silica column chromatography (eluent = hexanes/ethyl acetate, 8:1) in 67% yield (16.9 mg, >20:1 dr) as a white solid (mp 192–193 °C). ^1^H NMR (700 MHz, CDCl_3_): δ 6.77 (dd, *J* = 11.4, 5.9 Hz, 1H), 6.71 (dd, *J* = 6.2, 2.0 Hz, 1H), 6.60 (dd, *J* = 11.4, 6.2 Hz, 1H), 6.29 (ddd, *J* = 9.8, 5.9, 2.0 Hz, 1H), 5.57 (dd, *J* = 9.8, 4.2 Hz, 1H), 3.40 (dd, *J* = 8.3, 4.0 Hz, 1H), 3.10 (dd, *J* = 18.5, 8.3 Hz, 1H), 2.95 (dd, *J* = 18.5, 4.0 Hz, 1H), 2.76 (ddd, *J* = 4.2, 2.0, 2.0 Hz, 1H), 1.66 (s, 3H). ^13^C{^1^H} NMR (176 MHz, CDCl_3_): δ 171.6, 134.9, 129.0, 128.5, 127.3, 125.9, 121.4, 114.2, 112.5, 91.7, 55.6, 50.8, 41.5, 32.8, 26.3. HRMS (ESI-TOF) *m*/*z*: [M + Na]^+^ calcd for C_15_H_12_N_2_O_2_Na, 275.0796; found, 275.0798.

#### (3aR*,9aS*,9bR*)-9*b*-Ethyl-2-oxo-3,3*a*,9*a*,9*b*-tetrahydroazuleno[1,2-*b*]furan-4,4(2*H*)-dicarbonitrile (**3b**)

Following the general procedure, pure product **3b** was isolated after flash silica column chromatography (eluent = hexanes/ethyl acetate, 8:1) in 65% yield (17.3 mg, >20:1 dr) as a white solid (mp 144–145 °C). ^1^H NMR (700 MHz, CDCl_3_): δ 6.79 (dd, *J* = 11.1, 5.9 Hz, 1H), 6.70 (dd, *J* = 6.1, 1.9 Hz, 1H), 6.61 (dd, *J* = 11.2, 6.1 Hz, 1H), 6.27 (ddd, *J* = 9.7, 5.9, 1.9 Hz, 1H), 5.57 (dd, *J* = 9.7, 4.4 Hz, 1H), 3.43 (dd, *J* = 8.4, 3.6 Hz, 1H), 3.08 (dd, *J* = 18.6, 8.4 Hz, 1H), 2.95 (dd, *J* = 18.6, 3.5 Hz, 1H), 2.73 (ddd, *J* = 4.2, 1.9, 1.9 Hz, 1H), 1.91 (dq, *J* = 14.6, 7.3 Hz, 1H), 1.82 (dq, *J* = 14.7, 7.4 Hz, 1H), 1.07 (t, *J* = 7.3 Hz, 3H). ^13^C{^1^H} NMR (176 MHz, CDCl_3_): δ 171.9, 135.0, 128.9, 128.4, 126.7, 125.4, 122.0, 114.1, 112.6, 94.5, 54.2, 49.2, 41.7, 33.0, 32.9, 8.1. HRMS (ESI-TOF) *m*/*z*: [M + Na]^+^ calcd for C_16_H_14_N_2_O_2_Na, 289.0953; found, 289.0955.

#### (3aR*,9aS*,9bR*)-2-Oxo-9*b*-propyl-3,3*a*,9*a*,9*b*-tetrahydroazuleno[1,2-*b*]furan-4,4(2*H*)-dicarbonitrile (**3c**)

Following the general procedure, pure product **3c** was isolated after flash silica column chromatography (eluent = hexanes/ethyl acetate, 8:1) in 75% yield (21.0 mg, >20:1 dr) as a yellow oil. ^1^H NMR (700 MHz, CDCl_3_): δ 6.78 (dd, *J* = 11.2, 5.9 Hz, 1H), 6.69 (dd, *J* = 6.0, 1.9 Hz, 1H), 6.61 (dd, *J* = 11.0, 5.9 Hz, 1H), 6.27 (ddd, *J* = 9.7, 5.9, 1.9 Hz, 1H), 5.56 (ddd, *J* = 9.8, 4.4, 0.9 Hz, 1H), 3.42 (dd, *J* = 8.3, 3.3 Hz, 1H), 3.08 (dd, *J* = 18.6, 8.3 Hz, 1H), 2.95 (dd, *J* = 18.6, 3.3 Hz, 1H), 2.74 (ddd, *J* = 4.2, 2.0, 2.0 Hz, 1H), 1.86–1.79 (m, 1H), 1.76–1.71 (m, 1H), 1.63–1.56 (m, 2H), 0.96 (t, *J* = 7.3 Hz, 3H). ^13^C{^1^H} NMR (176 MHz, CDCl_3_): δ 171.9, 135.0, 129.0, 128.4, 126.7, 125.3, 122.0, 114.1, 112.5, 94.2, 54.7, 49.5, 42.2, 33.0, 29.8, 17.2, 14.2. HRMS (ESI-TOF) *m*/*z*: [M + Na]^+^ calcd for C_17_H_16_N_2_O_2_Na, 303.1109; found, 303.1113.

#### (3aR*,9aS*,9bR*)-9*b*-Hexyl-2-oxo-3,3*a*,9*a*,9*b*-tetrahydroazuleno[1,2-*b*]furan-4,4(2*H*)-dicarbonitrile (**3d**)

Following the general procedure, pure product **3d** was isolated after flash silica column chromatography (eluent = hexanes/ethyl acetate, 8:1) in 59% yield (19.0 mg, >20:1 dr) as a yellow oil. ^1^H NMR (700 MHz, CDCl_3_): δ 6.79 (dd, *J* = 11.2, 5.9 Hz, 1H), 6.70 (dd, *J* = 5.8, 2.2 Hz, 1H), 6.61 (dd, *J* = 11.9, 6.1 Hz, 1H), 6.27 (ddd, *J* = 9.7, 5.9, 1.9 Hz, 1H), 5.56 (dd, *J* = 9.7, 4.4 Hz, 1H), 3.42 (dd, *J* = 8.3, 3.4 Hz, 1H), 3.08 (dd, *J* = 18.6, 8.3 Hz, 1H), 2.95 (dd, *J* = 18.6, 3.4 Hz, 1H), 2.73 (ddd, *J* = 4.1, 1.9, 1.9 Hz, 1H), 1.87–1.80 (m, 1H), 1.79–1.72 (m, 1H), 1.37–1.26 (m, 8H), 0.87 (t, *J* = 7.1 Hz, 3H). ^13^C{^1^H} NMR (176 MHz, CDCl_3_): δ 171.9, 135.0, 129.0, 128.4, 126.8, 125.4, 122.1, 114.1, 112.6, 94.2, 54.6, 49.5, 40.1, 33.0, 31.6, 29.9, 29.3, 23.7, 22.6, 14.1. . HRMS (ESI-TOF) *m*/*z*: [M + Na]^+^ calcd for C_20_H_22_N_2_O_2_Na, 345.1579; found, 345.1581.

#### (3aR*,9aS*,9bR*)-9*b*-Isopropyl-2-oxo-3,3*a*,9*a*,9*b*-tetrahydroazuleno[1,2-*b*]furan-4,4(2*H*)-dicarbonitrile (**3e**)

Following the general procedure, pure product **3e** was isolated after flash silica column chromatography (eluent = hexanes/ethyl acetate, 8:1) in 43% yield (12.1 mg, >20:1 dr) as a white solid (mp 100–101 °C). ^1^H NMR (700 MHz, CDCl_3_): δ 6.82 (dd, *J* = 11.0, 5.9 Hz, 1H), 6.69 (dd, *J* = 6.0, 1.8 Hz, 1H), 6.63 (dd, *J* = 11.1, 6.0 Hz, 1H), 6.27 (ddd, *J* = 9.6, 5.9, 1.8 Hz, 1H), 5.60 (dd, *J* = 9.6, 4.5 Hz, 1H), 3.55 (dd, *J* = 8.4, 2.6 Hz, 1H), 3.08 (dd, *J* = 18.7, 8.4 Hz, 1H), 2.97 (dd, *J* = 18.8, 2.6 Hz, 1H), 2.70 (ddd, *J* = 4.3, 1.8, 1.8 Hz, 1H), 1.96 (hept, *J* = 6.7 Hz, 1H), 1.07 (d, *J* = 6.7 Hz, 3H), 1.01 (d, *J* = 6.8 Hz, 3H). ^13^C{^1^H} NMR (176 MHz, CDCl_3_): δ 172.1, 135.2, 128.8, 128.3, 126.3, 124.5, 122.7, 114.0, 112.6, 97.2, 52.6, 47.5, 42.4, 36.7, 33.3, 17.3, 17.0. HRMS (ESI-TOF) *m*/*z*: [M + H]^+^ calcd for C_17_H_16_N_2_O_2_H, 281.1290; found, 281.1296.

#### (3aR*,9aS*,9bR*)-9*b*-Benzyl-2-oxo-3,3*a*,9*a*,9*b*-tetrahydroazuleno[1,2-*b*]furan-4,4(2*H*)-dicarbonitrile (**3f**)

Following the general procedure, pure product **3f** was isolated after flash silica column chromatography (eluent = hexanes/ethyl acetate, 8:1) in 60% yield (19.7 mg, >20:1 dr) as a white solid (mp 209–210 °C). ^1^H NMR (700 MHz, CDCl_3_): δ 7.44–7.34 (m, 3H), 7.32–7.25 (m, 2H), 6.79 (dd, *J* = 11.1, 5.9 Hz, 1H), 6.72 (dd, *J* = 6.1, 1.9 Hz, 1H), 6.63 (dd, *J* = 11.2, 6.1 Hz, 1H), 6.27 (ddd, *J* = 9.7, 5.9, 1.9 Hz, 1H), 5.55 (dd, *J* = 9.8, 4.3 Hz, 1H), 3.47 (dd, *J* = 8.6, 3.9 Hz, 1H), 3.35 (d, *J* = 14.5 Hz, 1H), 2.96 (d, *J* = 14.5 Hz, 1H), 2.83 (ddd, *J* = 4.1, 1.9, 1.9 Hz, 1H), 2.66 (dd, *J* = 18.4, 3.9 Hz, 1H), 2.22 (dd, *J* = 18.4, 8.6 Hz, 1H). ^13^C{^1^H} NMR (176 MHz, CDCl_3_): δ 171.9, 135.0, 133.5, 130.5 (2C), 129.4 (2C), 128.5, 128.5, 128.3, 127.0, 125.6, 121.6, 114.2, 112.7, 93.4, 53.1, 50.7, 45.4, 41.8, 33.3. HRMS (ESI-TOF) *m*/*z*: [M + Na]^+^ calcd for C_21_H_16_N_2_O_2_Na, 351.1109; found, 351.1111.

#### (3aR*,9aS*,9bR*)-9*b*-Allyl-2-oxo-3,3*a*,9*a*,9*b*-tetrahydroazuleno[1,2-*b*]furan-4,4(2*H*)-dicarbonitrile (**3g**)

Following the general procedure, pure product **3g** was isolated after flash silica column chromatography (eluent = hexanes/ethyl acetate, 8:1) in 43% yield (11.9 mg, >20:1 dr) as a white solid (mp 121–122 °C). ^1^H NMR (700 MHz, CDCl_3_): δ 6.78 (dd, *J* = 11.2, 5.9 Hz, 1H), 6.70 (dd, *J* = 6.0, 1.9 Hz, 1H), 6.61 (dd, *J* = 11.2, 5.7 Hz, 1H), 6.28 (ddd, *J* = 9.8, 5.9, 2.0 Hz, 1H), 5.83–5.76 (m, 1H), 5.54 (dd, *J* = 9.8, 4.3 Hz, 1H), 5.33–5.26 (m, 2H), 3.50 (dd, *J* = 8.6, 4.3 Hz, 1H), 3.06 (dd, *J* = 18.5, 8.6 Hz, 1H), 2.92 (dd, *J* = 18.5, 4.3 Hz, 1H), 2.76 (ddd, *J* = 4.1, 2.0, 2.0 Hz, 1H), 2.66–2.56 (m, 2H). ^13^C{^1^H} NMR (176 MHz, CDCl_3_): δ 171.8, 134.9, 129.8, 128.5, 128.5, 127.0, 125.7, 122.4, 121.5, 114.1, 112.7, 93.3, 53.2, 49.4, 43.6, 41.5, 33.2. HRMS (ESI-TOF) *m*/*z*: [M + Na]^+^ calcd for C_17_H_14_N_2_O_2_Na, 301.0953; found, 301.0949.

## Supporting Information Available

Full account of screening results, X-ray structure, and copies of ^1^H and ^13^C NMR spectra (PDF)

## Accession Codes

CCDC 2107237 contains the supplementary crystallographic data for this paper. These data can be obtained free of charge via www.ccdc.cam.ac.uk/data_request/cif, or by emailing data_request@ccdc.cam.ac.uk, or by contacting The Cambridge Crystallographic Data Centre, 12 Union Road, Cambridge CB2 1EZ, UK; fax: +44 1223 336033.
