# Supplementary material for: 5-Substituted-furan-2(3H)-ones in [8 + 2]-Cycloaddition with 8,8-Dicyanoheptafulvene
Source: J Org Chem. 2022 Mar 29;87(8):5296–302. doi: 10.1021/acs.joc.2c00101 (PMC9016758; doi:10.1021/acs.joc.2c00101)
Supplement: Supplementary file 1 — jo2c00101_si_001.pdf [file jo2c00101_si_001.pdf]

5-Substituted-furan-2(3*H*)-ones in [8+2]-cycloaddition with 8,8-dicyanoheptafulvene

Marta Romaniszyn,[a] Lesław Sieroń,[b] and Łukasz Albrecht[a,*]

[a] Institute of Organic Chemistry, Faculty of Chemistry, Lodz University of Technology

Żeromskiego 116, 90-924 Łódź, Poland

e-mail: lukasz.albrecht@p.lodz.pl

[b] Institute of General and Ecological Chemistry, Faculty of Chemistry, Lodz University of Technology, Żeromskiego 116, 90-924 Łódź, Poland

Table of contents

**1.**	Synthesis of (3a*R**,9a*S**,9b*R**)-9b-methyl-2-oxo-3,3a,9a,9b-tetrahydroazuleno[1,2-b]furan-4,4(2*H*)-dicarbonitrile **3a** on a 1 mmol scale	S2
**2.**	NMR data	S3
**3.**	HPLC traces for asymmetric attempts	S12
**4.**	Crystal and X ray data for **3a**	S14

1. Synthesis of (3aR*,9aS*,9bR*)-9b-methyl-2-oxo-3,3a,9a,9b-tetrahydroazuleno[1,2-b]furan-4,4(2*H*)-dicarbonitrile 3a on a 1 mmol scale

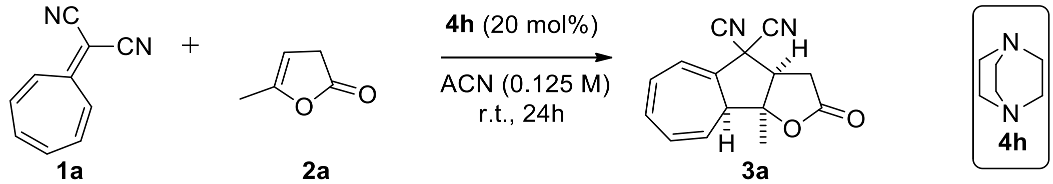

1a 2a 3a

Scheme S1

In an ordinary 25 mL round-bottom flask, equipped with a magnetic stirring bar and a septum 5-methyl-furane-2(3*H*)-one **2a** (2.0 equiv, 2 mmol, 89,8 µl), 8,8-dicyanoheptafulvene **1a** (1.0 equiv, 1 mmol, 154,2 mg) and catalyst **4h** (0.02 equiv., 0,2 mmol, 22,4 mg) were dissolved in acetonitrile (8 mL) and stirred for 24 h at room temperature. The reaction mixture was directly subjected to flash chromatography on silica gel (eluent: hexanes/ethyl acetate 8:1) to isolate pure product **3a** in 68% yield (171.5 mg, >20:1 dr) as a white solid (mp = 192-193 °C). Spectral data were in accordance to the reported in the manuscript. HRMS (ESI-TOF) m/z: [M + Na]+ Calcd for $C_{15}H_{12}N_2O_2Na$ 275.0796; Found 275.0798.

2. NMR data

5-Benzylfuran-2(3*H*)-one (2f)

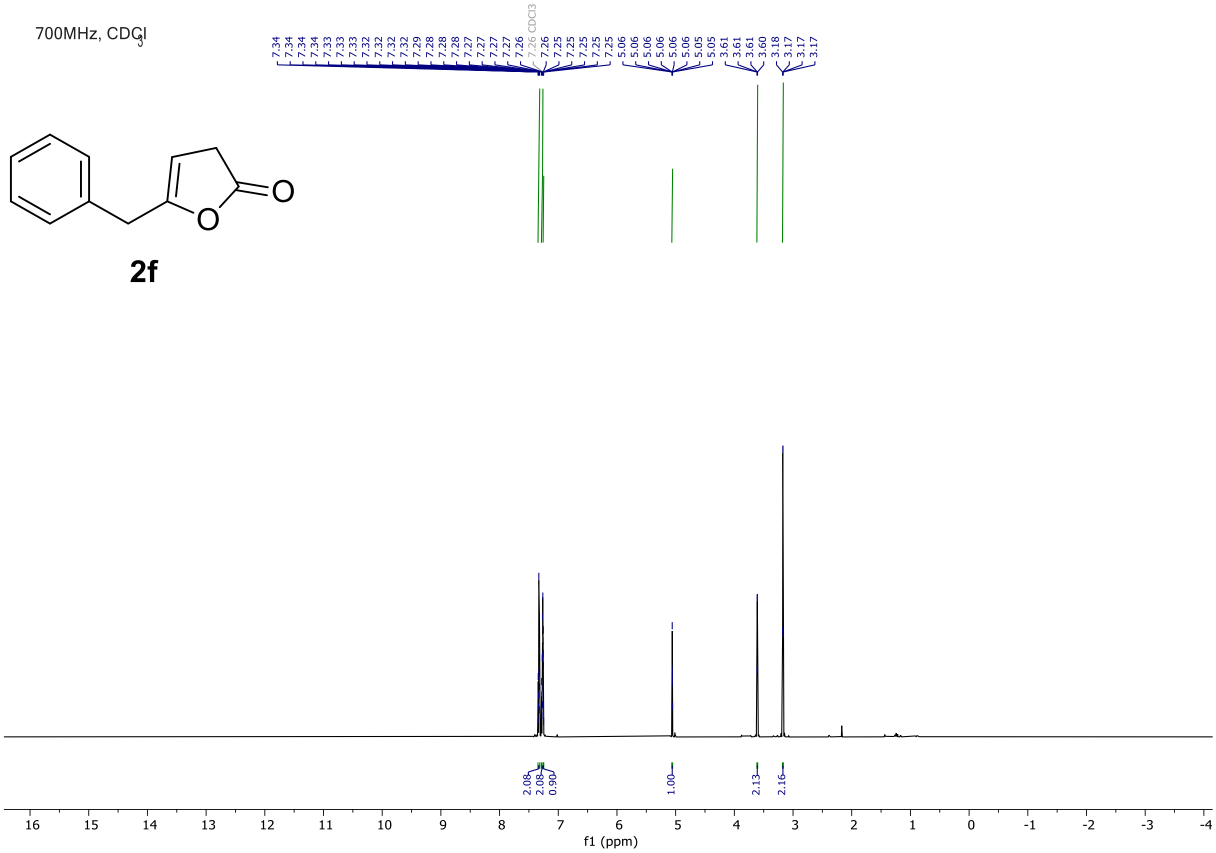

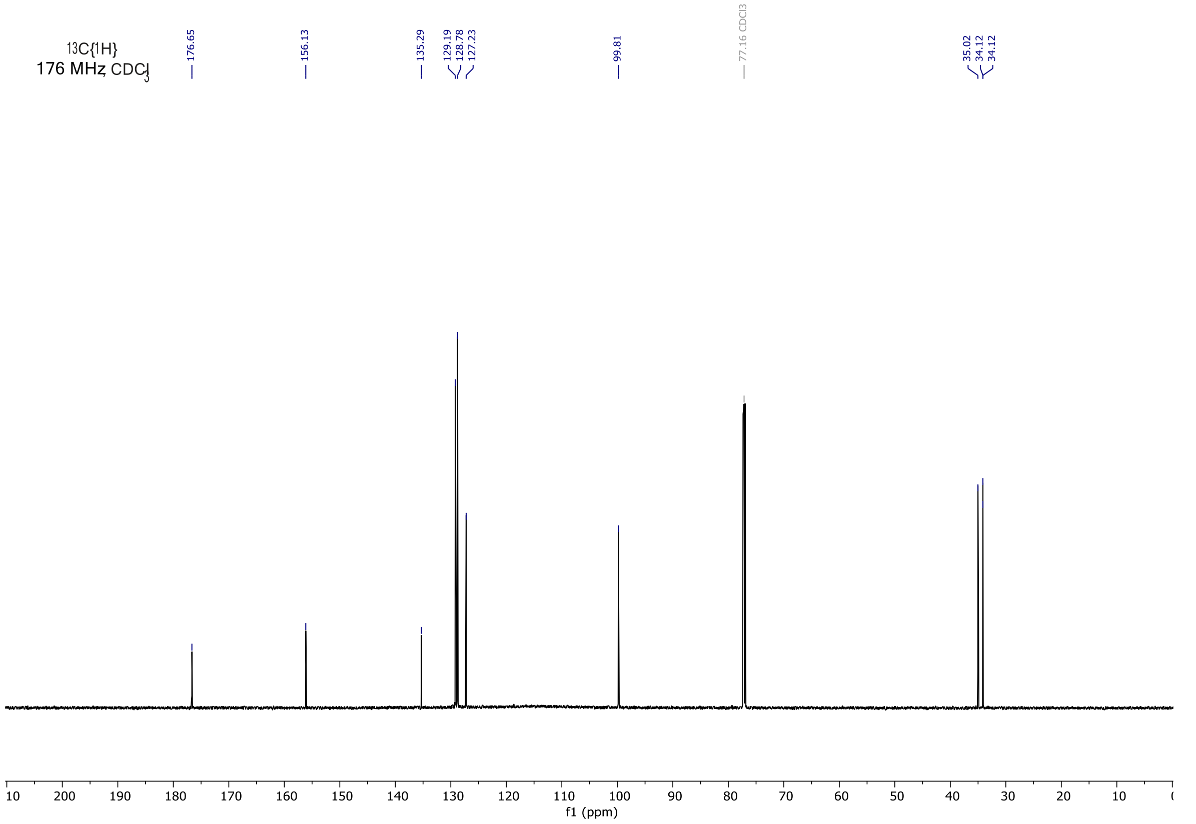

5-Allylfuran-2(3*H*)-one (2g)

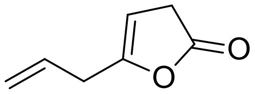

2g

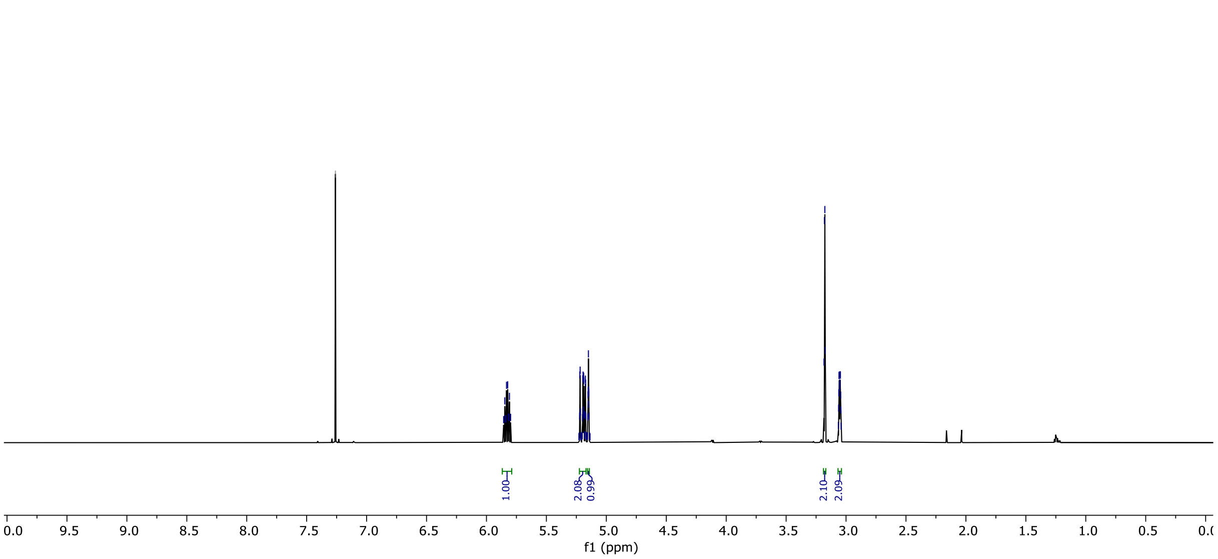

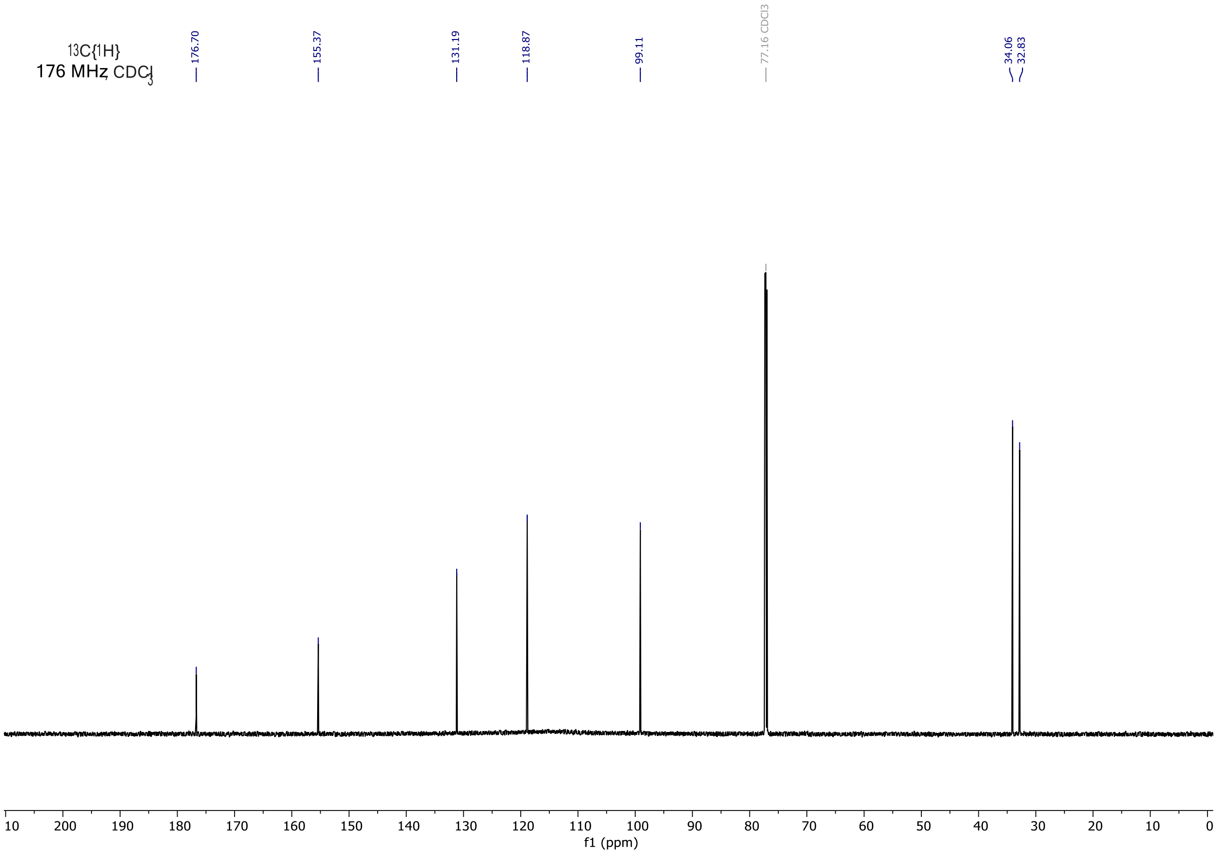

(3a*R**,9a*S**,9b*R**)-9b-Methyl-2-oxo-3,3a,9a,9b-tetrahydroazuleno[1,2-b]furan-4,4(2*H*)-dicarbonitrile (3a)

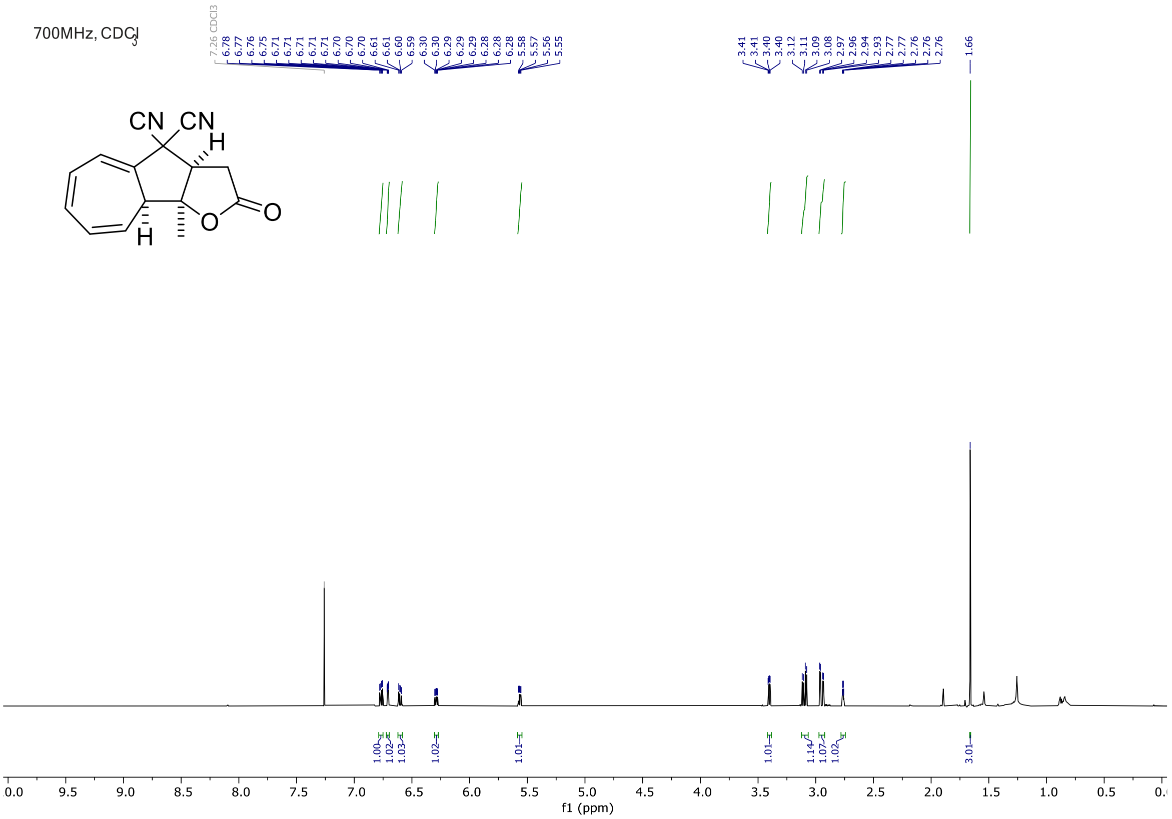

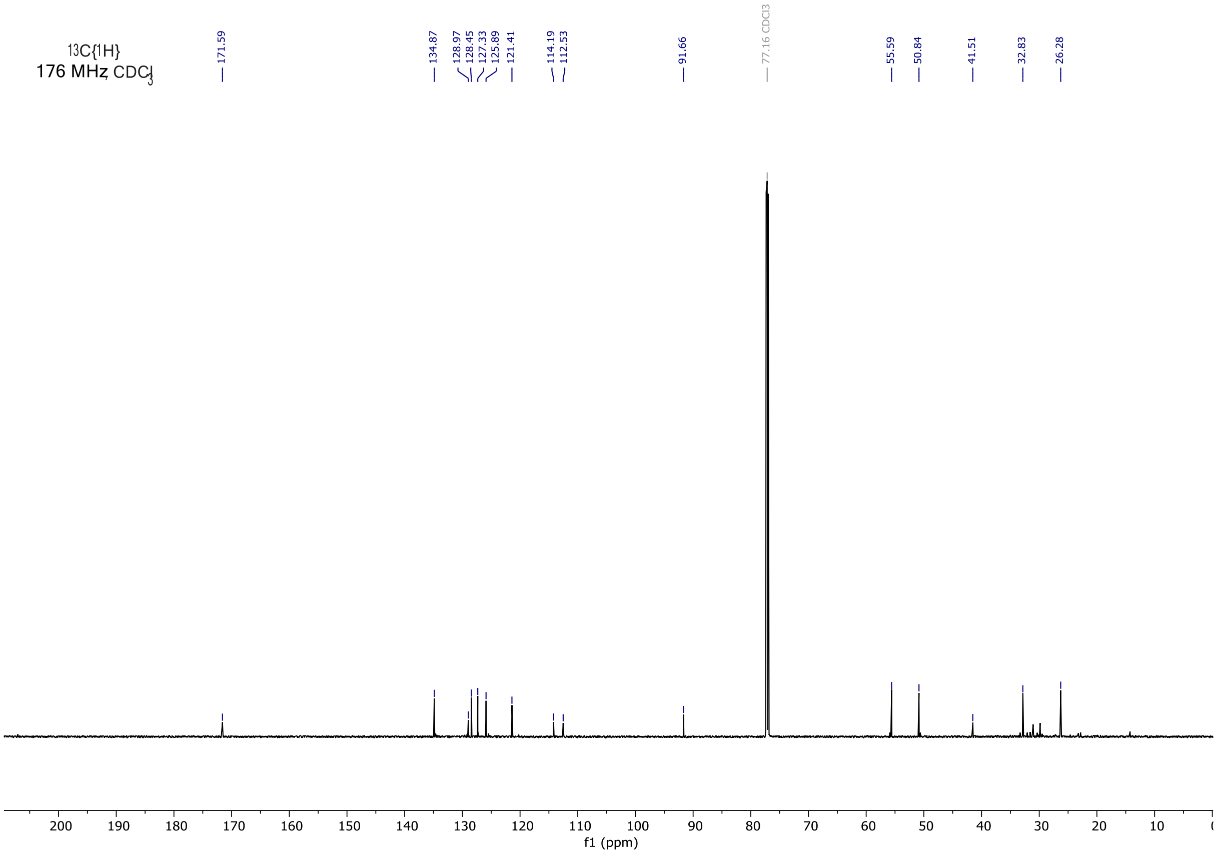

3a*R,9a*S**,9b*R**)-9b-Ethyl-2-oxo-3,3a,9a,9b-tetrahydroazuleno[1,2-b]furan-4,4(2*H*)-dicarbonitrile (3b)**

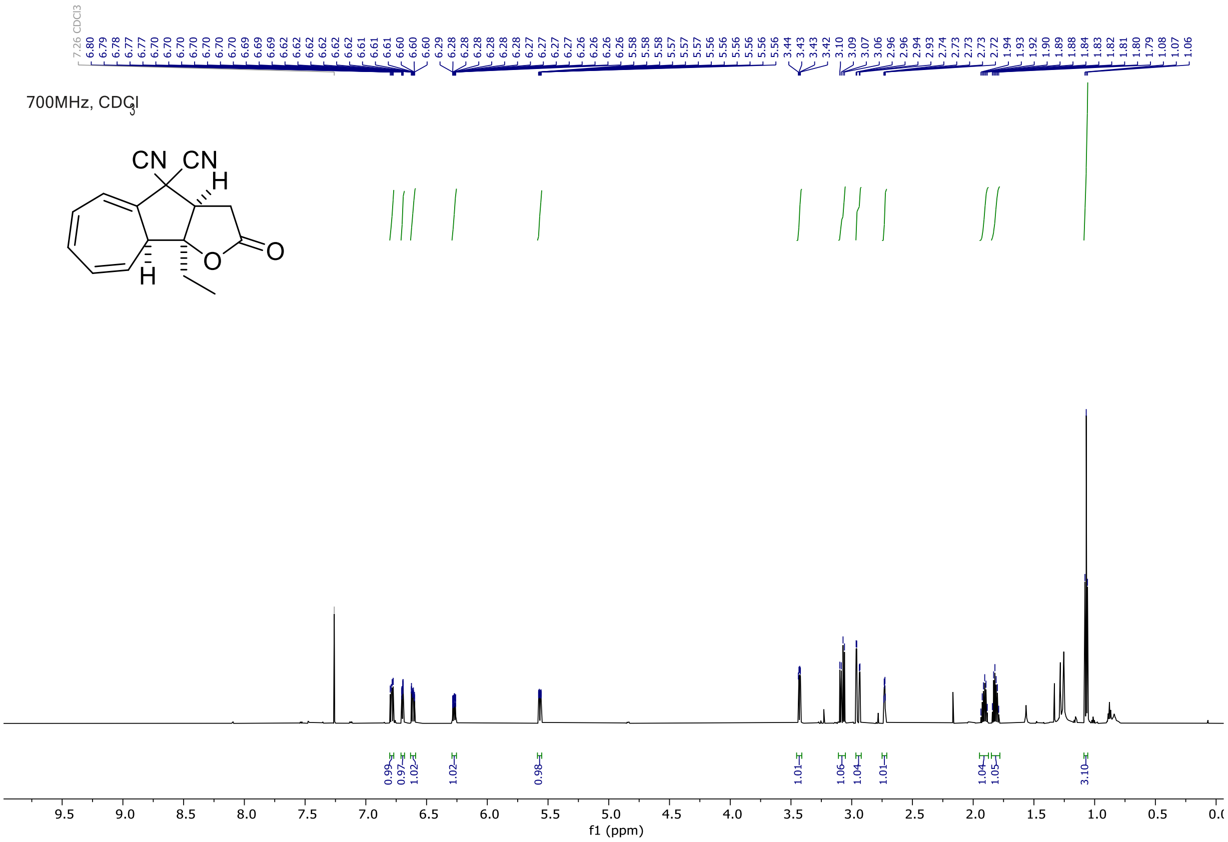

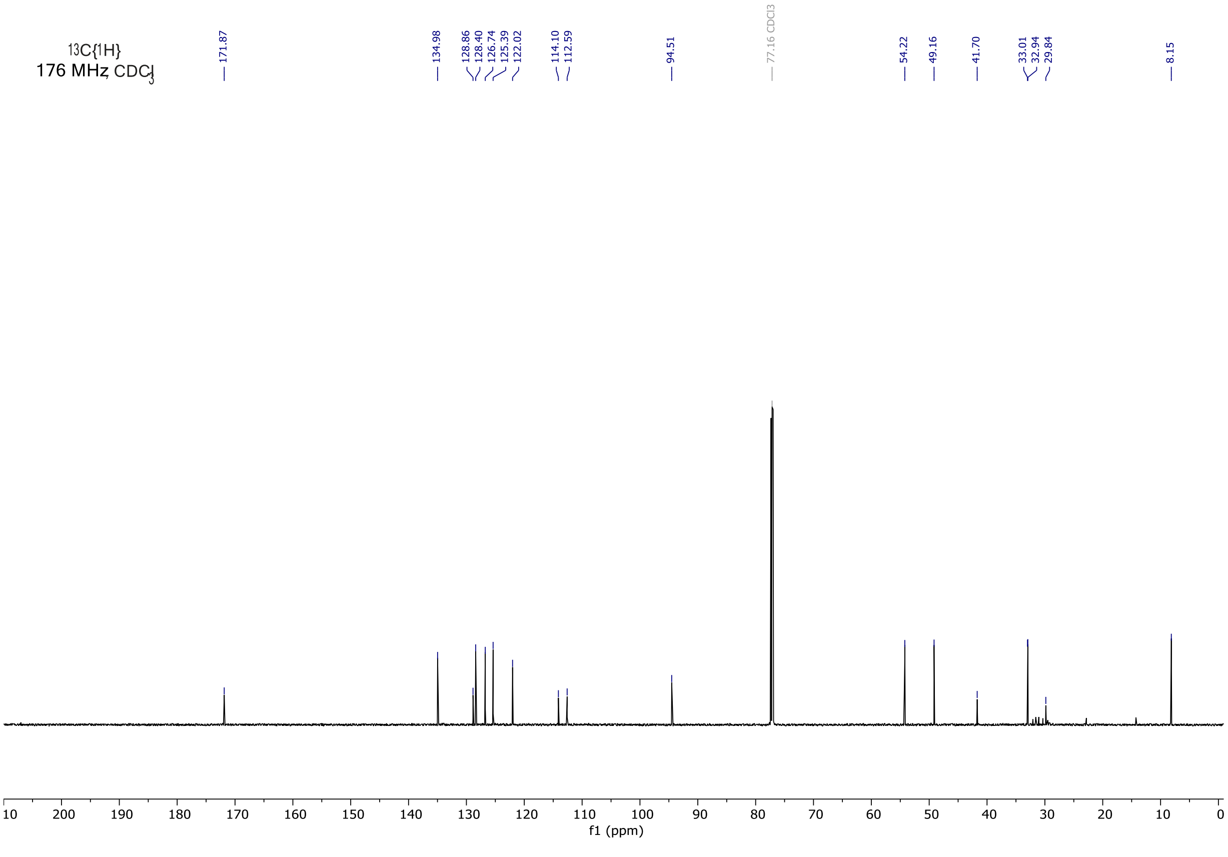

(3a*R,9a*S**,9b*R**)-2-oxo-9b-propyl-3,3a,9a,9b-tetrahydroazuleno[1,2-b]furan-4,4(2*H*)-dicarbonitrile (3c)**

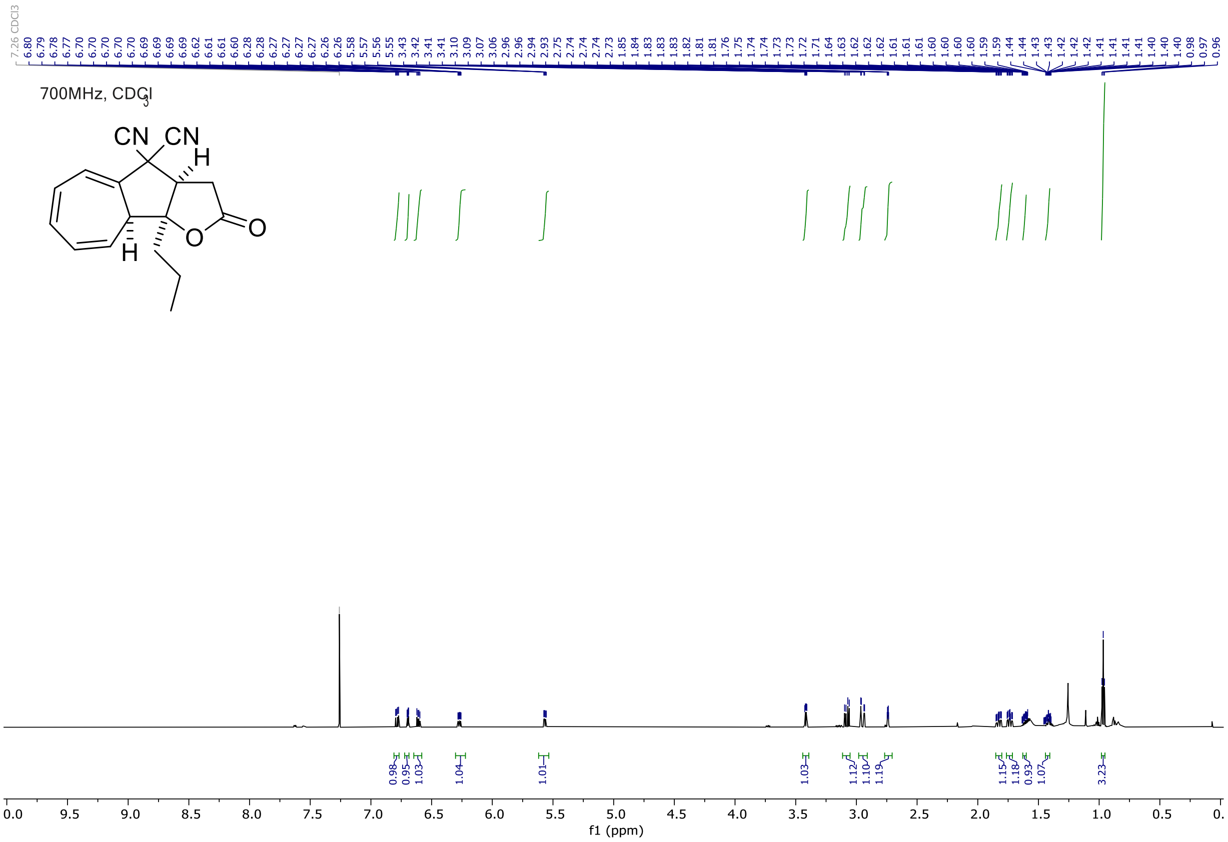

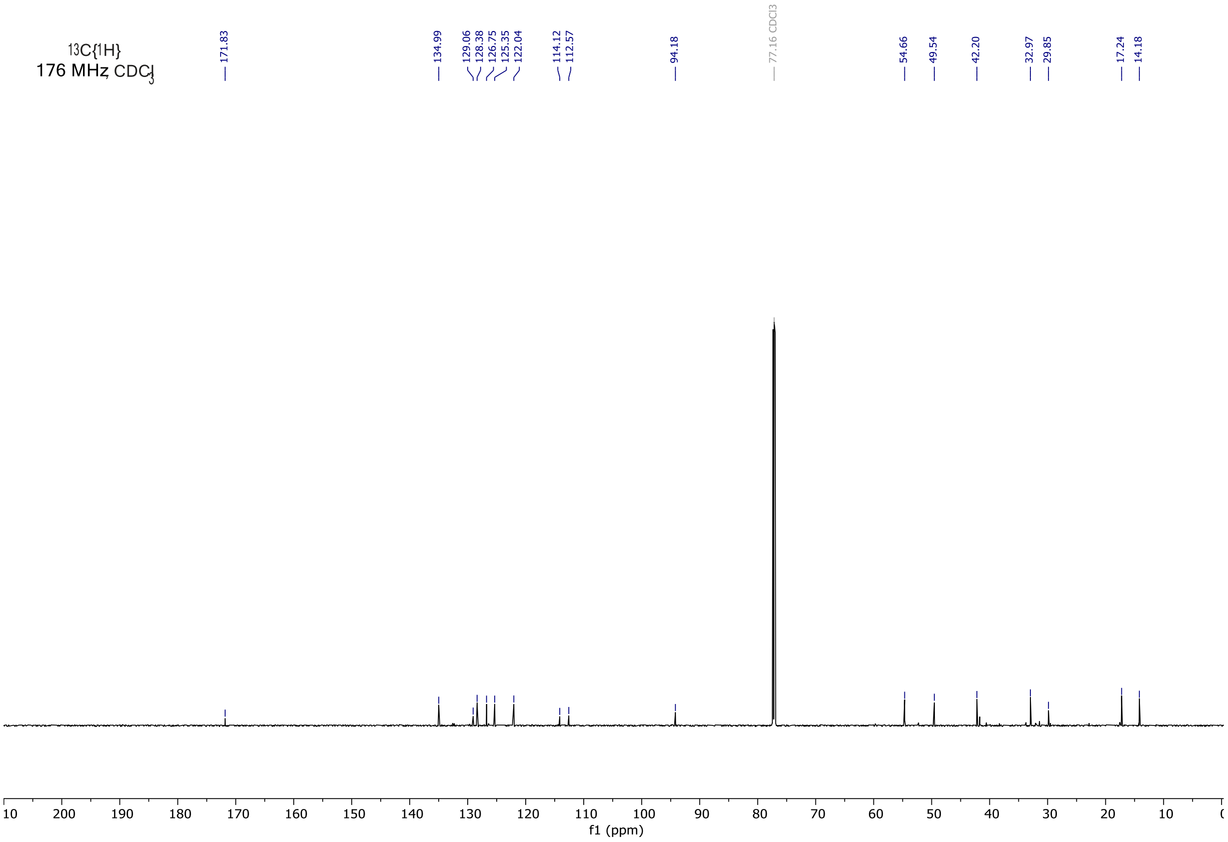

(3a*R**,9a*S**,9b*R**)-9b-hexyl-2-oxo-3,3a,9a,9b-tetrahydroazuleno[1,2-b]furan-4,4(2*H*)-dicarbonitrile (3d)

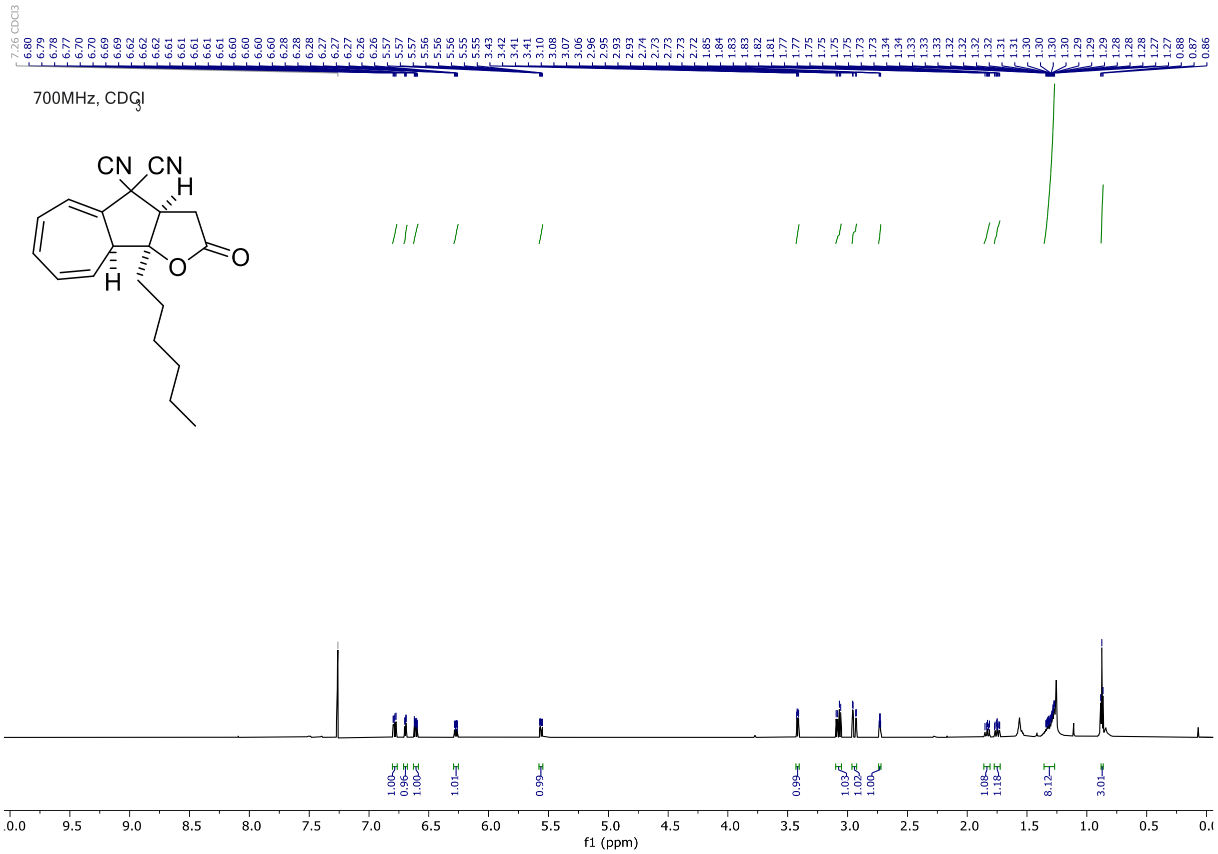

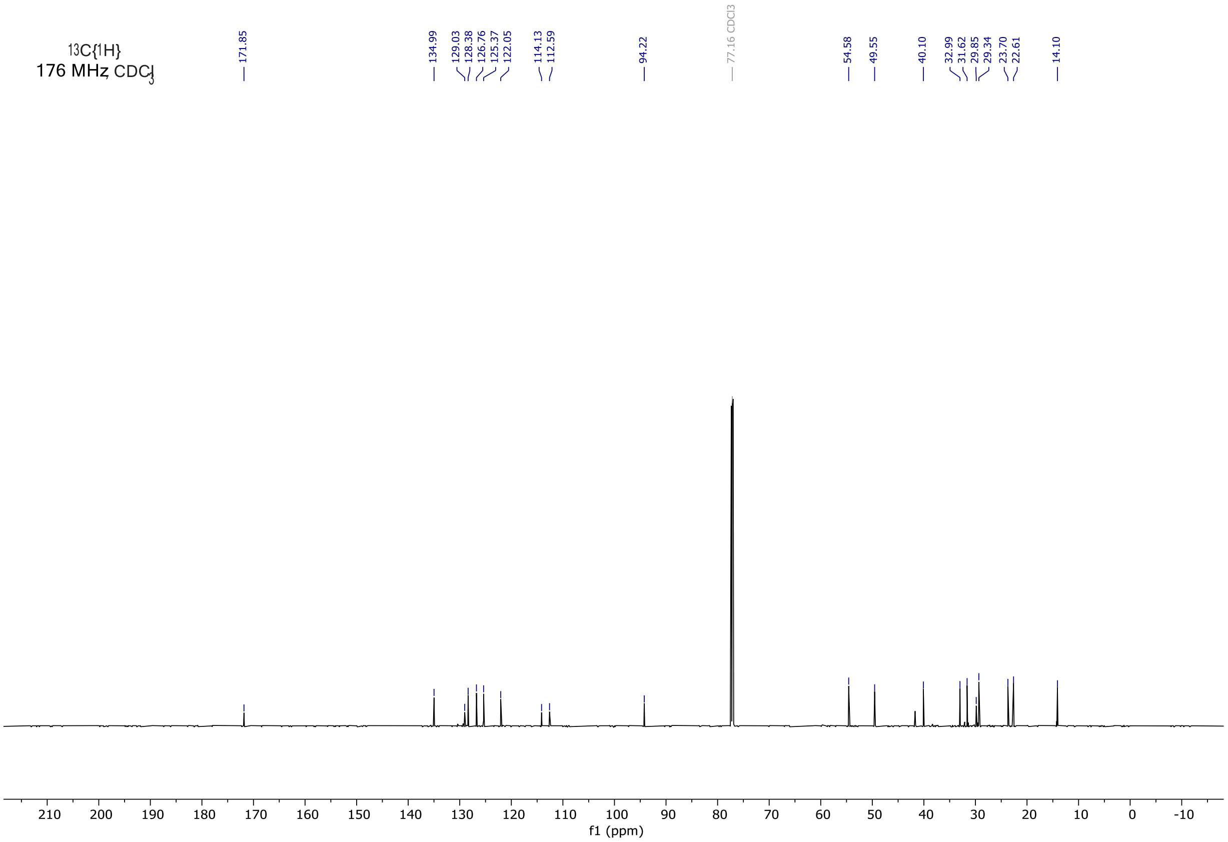

(3a*R**,9a*S**,9b*R**)-9b-isopropyl-2-oxo-3,3a,9a,9b-tetrahydroazuleno[1,2-b]furan-4,4(2*H*)-dicarbonitrile (3e)

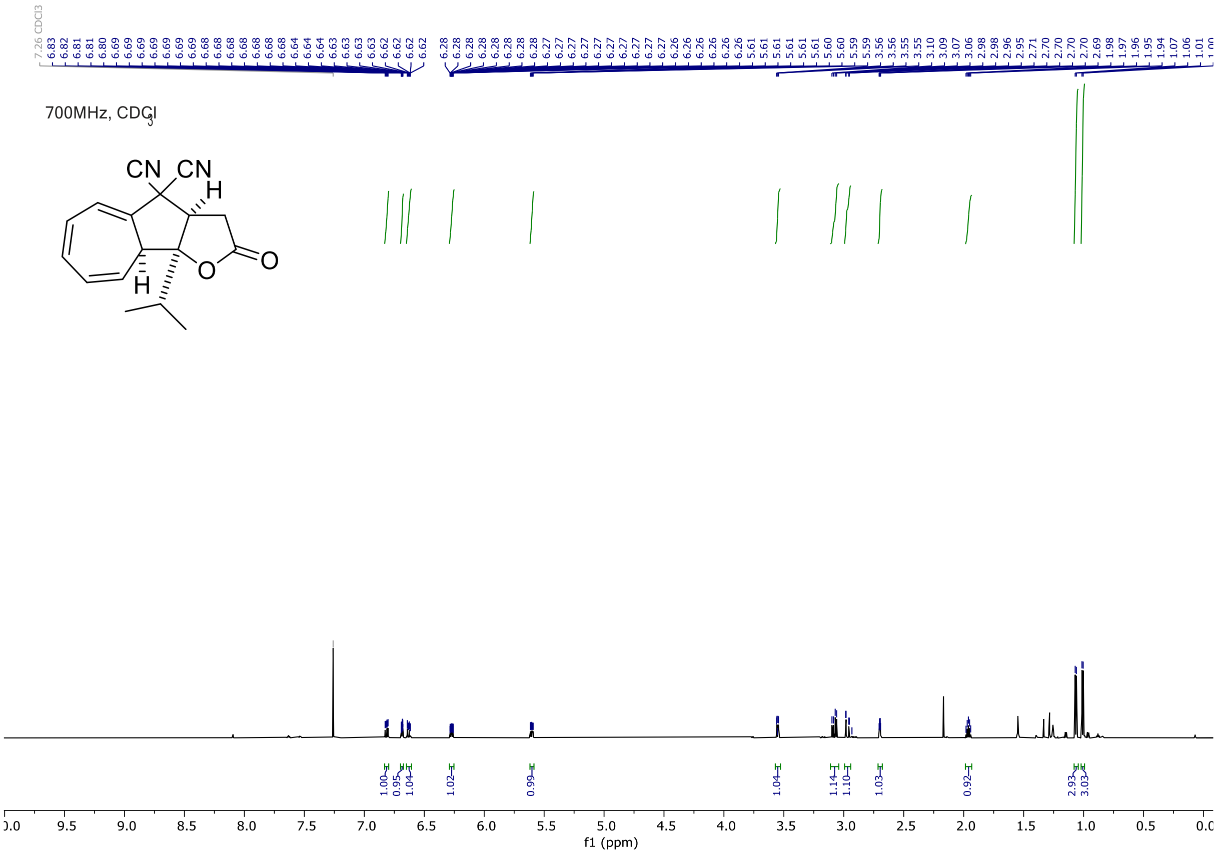

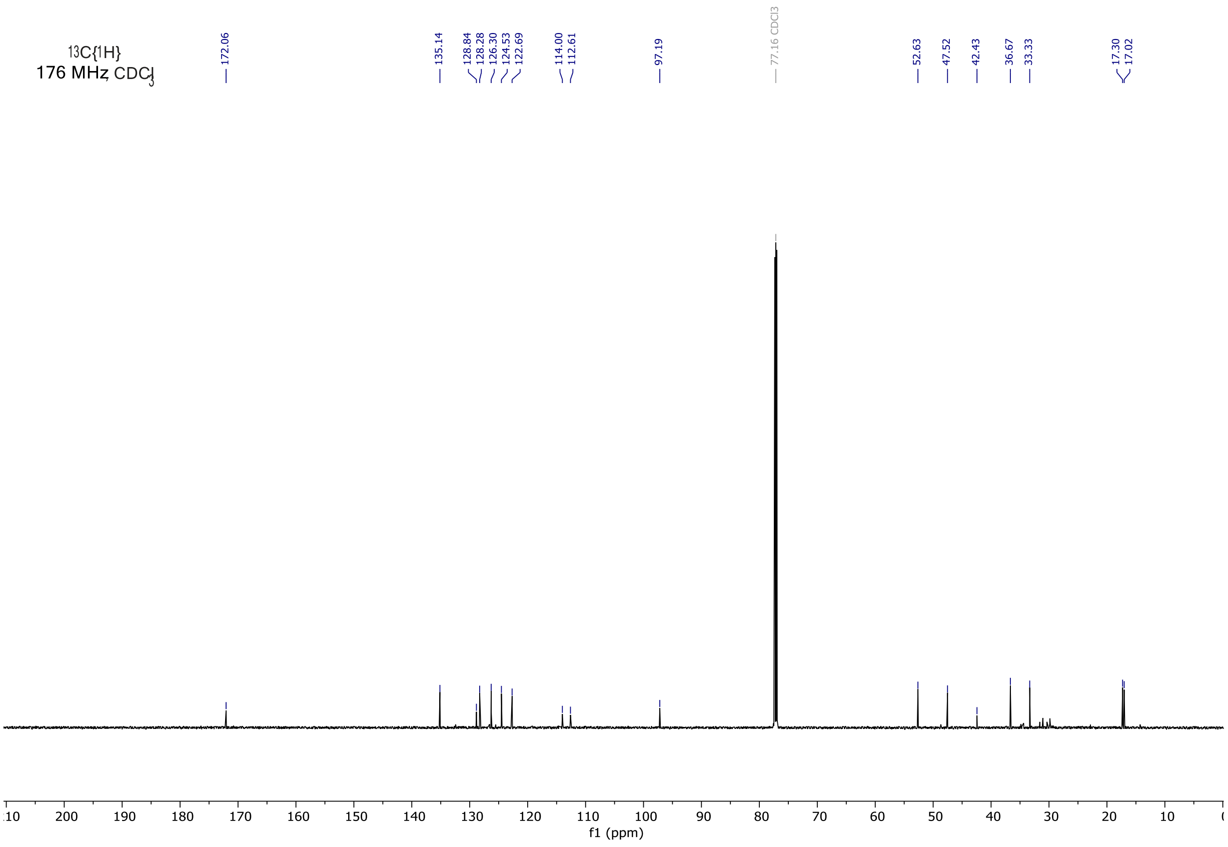

(3a*R,9a*S**,9b*R**)-9b-benzyl-2-oxo-3,3a,9a,9b-tetrahydroazuleno[1,2-b]furan-4,4(2*H*)-dicarbonitrile (3f)**

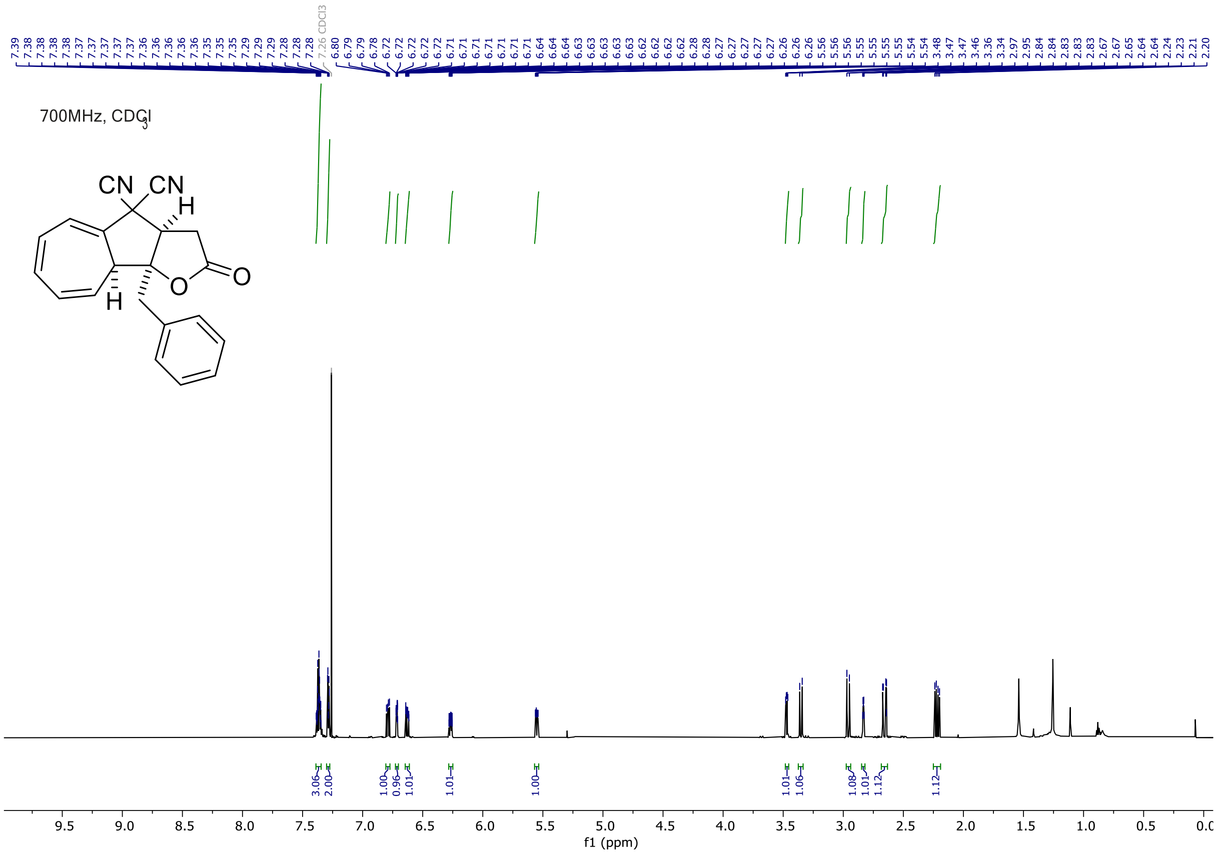

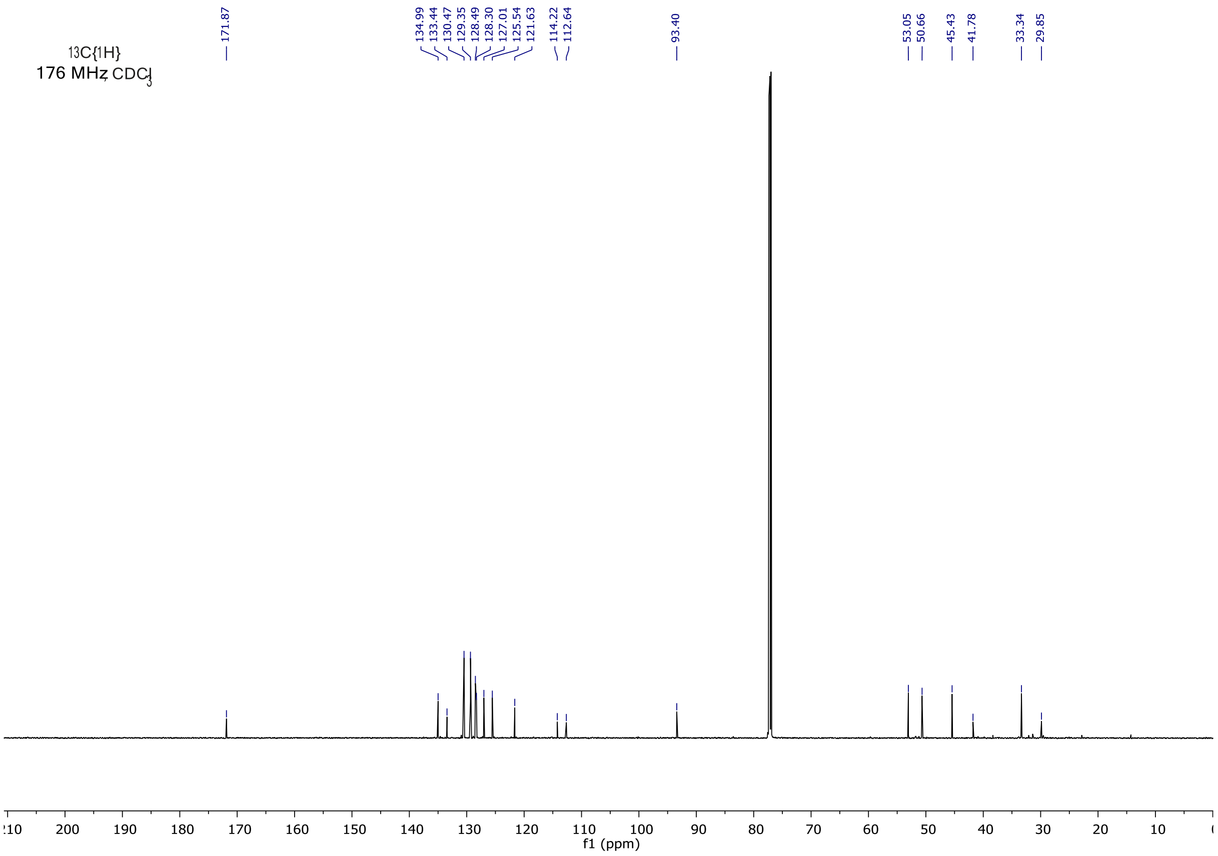

(3a*R**,9a*S**,9b*R**)-9b-allyl-2-oxo-3,3a,9a,9b-tetrahydroazuleno[1,2-b]furan-4,4(2*H*)-dicarbonitrile (3g)

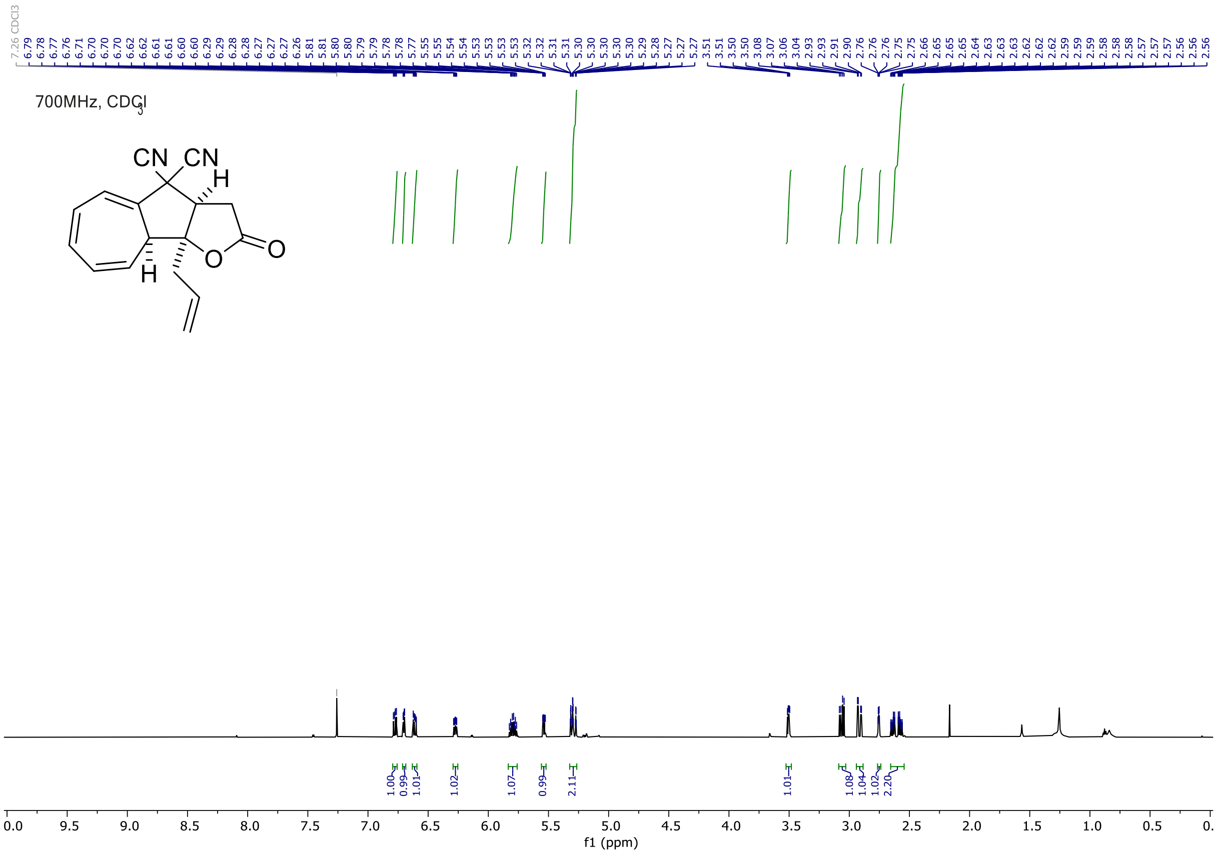

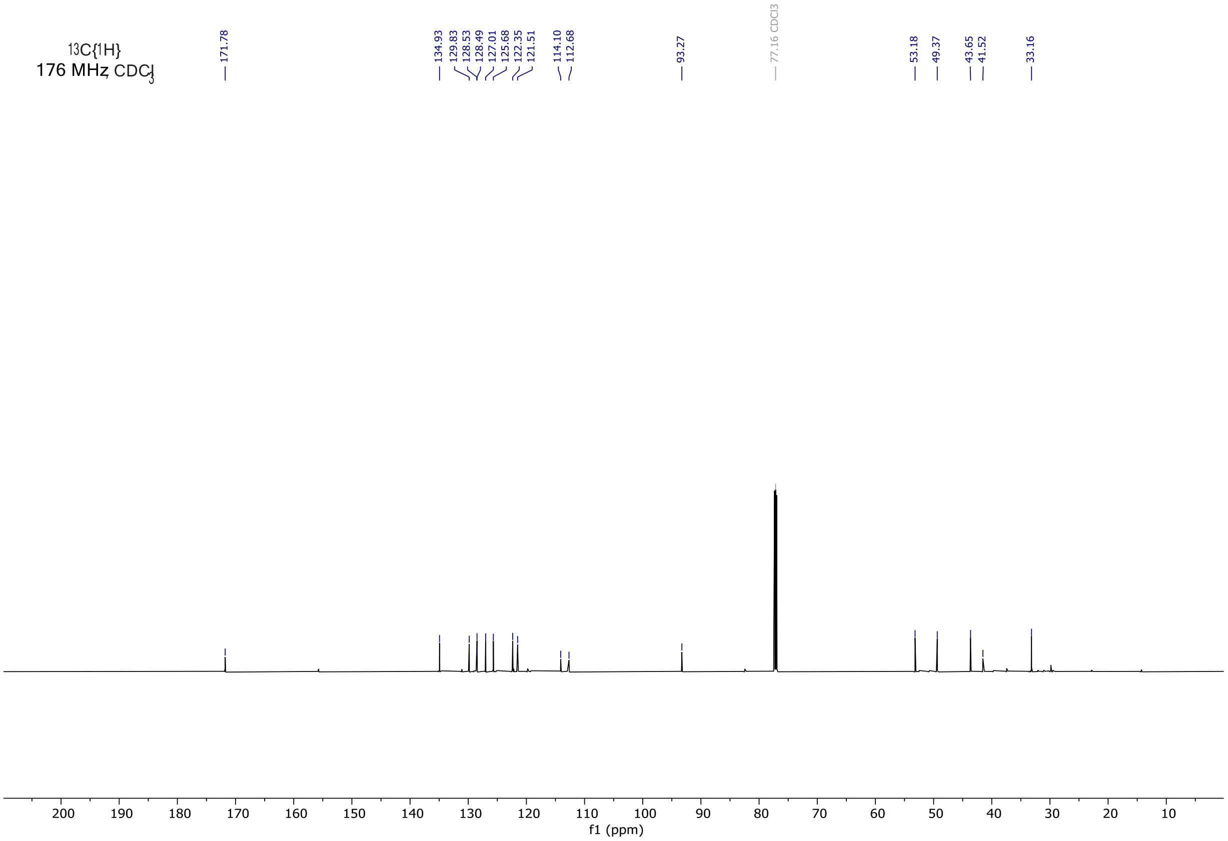

3. HPLC traces for asymmetric attempts

HPLC traces of the product *ent*-3a obtained in the synthesis with the use of catalyst **4i**

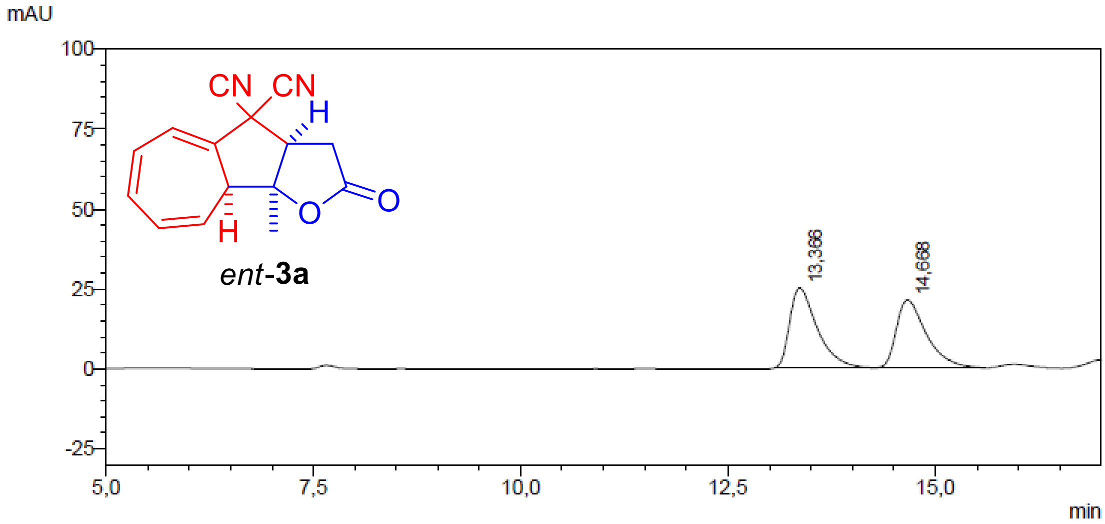

PDA Ch2 301nm

Peak#	Ret. Time	Area%
1	13,366	51,919
2	14,668	48,081
Total		100,000

HPLC traces of the product *ent*-3a obtained in the synthesis with the use of catalyst **4j**

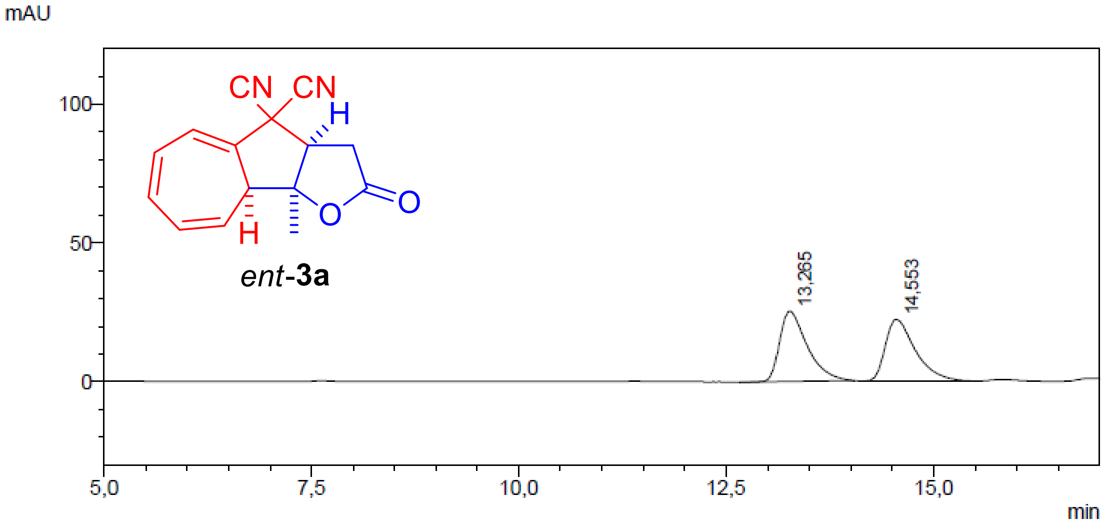

PDA Ch2 301nm

Peak#	Ret. Time	Area%
1	13,265	51,212
2	14,553	48,788
Total		100,000

HPLC traces of the product *ent*-3a obtained in the synthesis with the use of catalyst **4k**

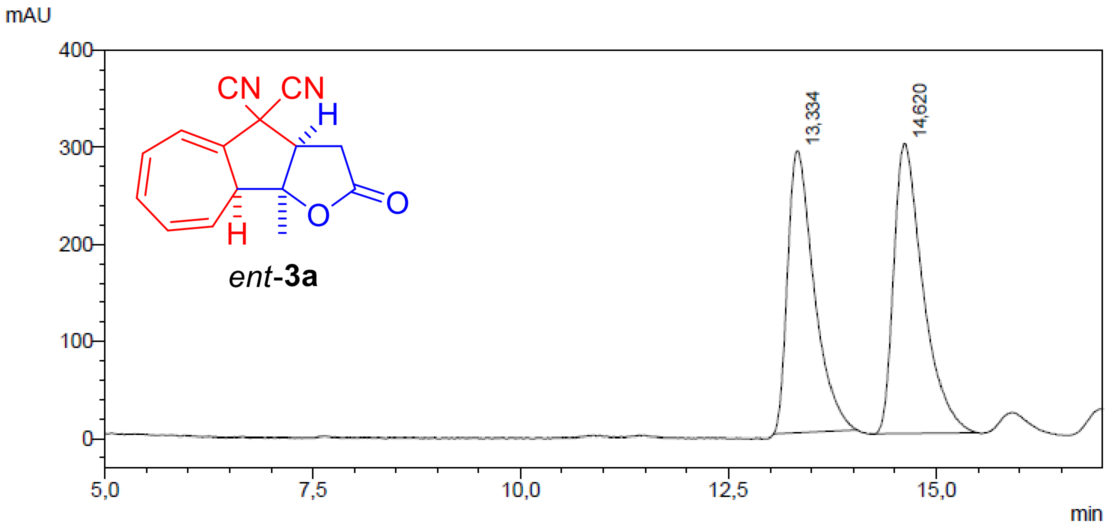

PDA Ch2 190nm

Peak#	Ret. Time	Area%
1	13,334	46,778
2	14,620	53,222
Total		100,000

HPLC traces of the product *ent*-3a obtained in the synthesis with the use of catalyst **4l**

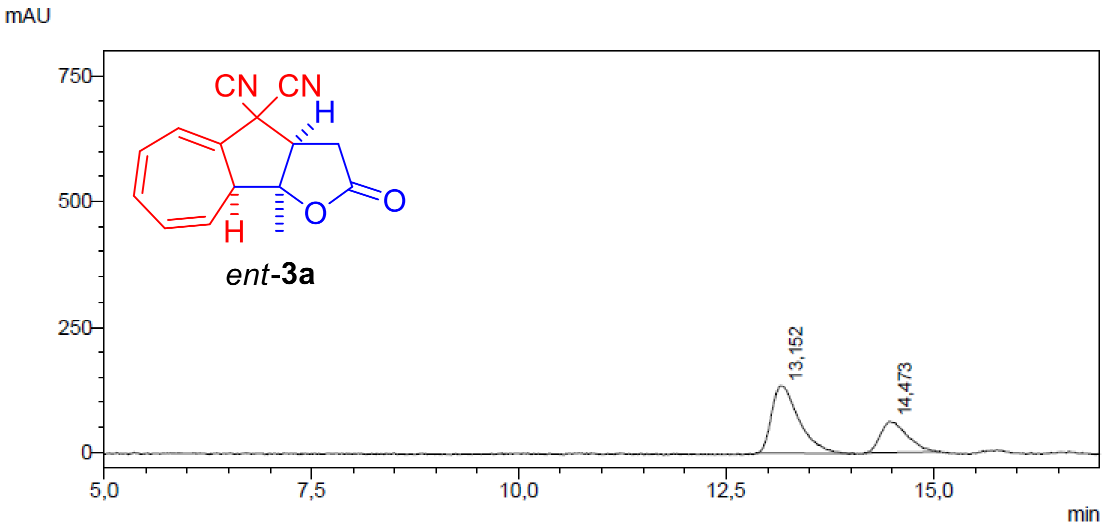

PDA Ch2 190nm

Peak#	Ret. Time	Area%
1	13,152	68,412
2	14,473	31,588
Total		100,000

4. Crystal and X ray data for 3a

The crystal structure of the compound **3a**, $C_{15}H_{12}N_2O_2$, was established by single-crystal X-ray diffraction at 100 K. The compound crystallizes (from hexane/Et_2O) as a racemate in the centrosymmetric monoclinic space group $P2_1/c$ (Z = 4) and the crystal structure consists of one crystallographically independent formula unit in the unit cell (Figure S1).

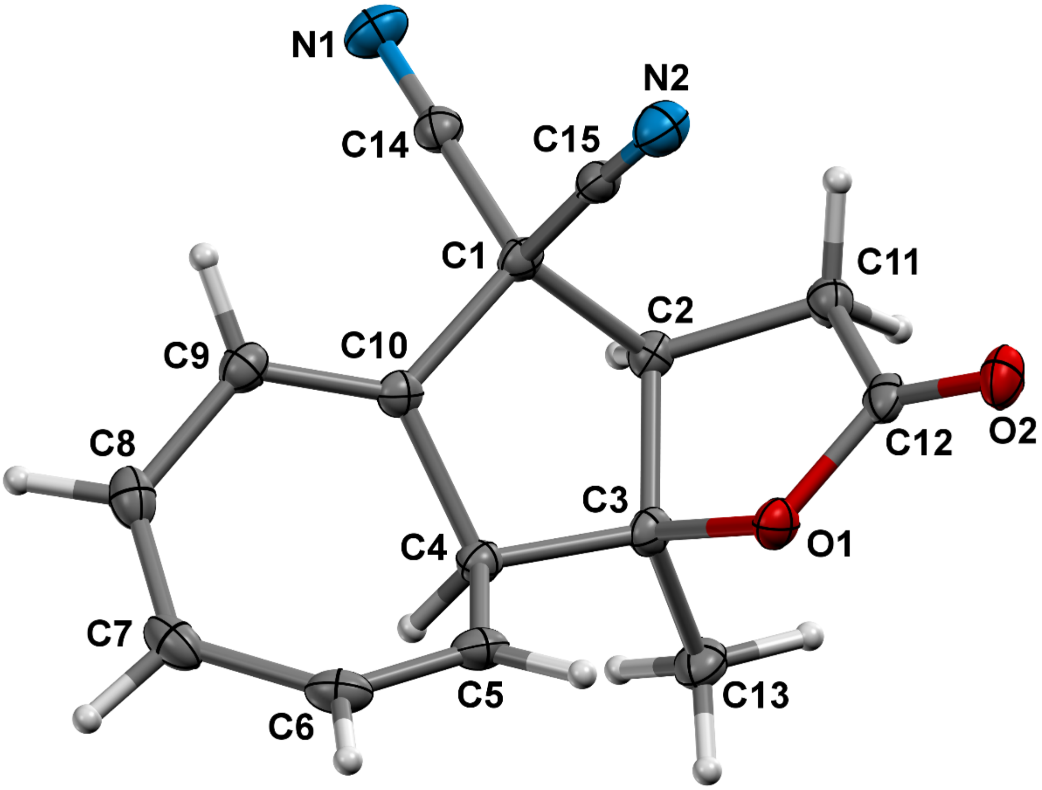

Figure S1. The molecular structure of the compound **3a** (one of the racemic enantiomers) at 100 K, with the atom labeling scheme. Displacement ellipsoids are shown at the 50% probability level. Hydrogen atoms are drawn with an arbitrary radius.

Single crystal X-ray diffraction data were collected at 100 K by the ω-scan technique using a RIGAKU XtaLAB Synergy, Dualflex, Pilatus 300K diffractometer[1] with PhotonJet micro-focus X-ray Source Cu-Kα (λ = 1.54184 Å). Data collection, cell refinement, data reduction and absorption correction were performed using CrysAlis PRO software.[2] The crystal structure was solved by using direct methods with the SHELXT 2018/2 program.[3] Atomic scattering factors were taken from the International Tables for X-ray Crystallography. Positional parameters of non-H-atoms were refined by a full-matrix least-squares method on F^2 with anisotropic thermal parameters by using the SHELXL 2018/3 program.[3] All hydrogen atoms were found from the difference Fourier maps and for further calculations they were positioned geometrically in

calculated positions (C–H = 0.95–1.00 Å) and constrained to ride on their parent atoms with isotropic displacement parameters set to 1.2-1.5 times the U_{eq} of the parent atom.

3a: Formula $C_{15}H_{12}N_2O_2$, orthorhombic, space group $P2_1/c$, Z = 4, unit cell constants a = 13.7399(1), b = 6.9570(1), c = 12.8835(1) Å, β = 95.051(1)°, V = 1226.73(2) Å^3. The integration of the data yielded a total of 41252 reflections with θ angles in the range of 6.47 to 66.59°, of which 2173 were independent (R_{int} = 2.74%), and 2104 were greater than 2σ(F^2). The final anisotropic full-matrix least-squares refinement on F^2 with 174 parameters converged at R_1 = 3.16% and wR_2 = 7.47% for all data. The largest peak in the final difference electron density synthesis was 0.285 e Å^{-3} and the largest hole was -0.188 e Å^{-3}. The goodness-of-fit was 1.035.

CCDC 2107237 contains the supplementary crystallographic data for this paper. These data can be obtained free of charge from The Cambridge Crystallographic Data Centre via www.ccdc.cam.ac.uk/structures

References

1. Rigaku OD. CrysAlis PRO. Rigaku Oxford Diffraction Ltd, Yarnton, Oxfordshire, England, **2019**.

2. Sheldrick, G. M. SHELXT - integrated space-group and crystal-structure determination. *Acta Cryst.* **2015**, *A71*, 3-8.

3. Sheldrick, G. M. Crystal structure refinement with SHELXL. *Acta Cryst.* **2015**, *C71*, 3-8.
